# Variability in the prevalence of depression among adults with chronic pain: UK Biobank analysis through clinical prediction models

**DOI:** 10.1186/s12916-024-03388-x

**Published:** 2024-04-19

**Authors:** Lingxiao Chen, Claire E Ashton-James, Baoyi Shi, Maja R Radojčić, David B Anderson, Yujie Chen, David B Preen, John L Hopper, Shuai Li, Minh Bui, Paula R Beckenkamp, Nigel K Arden, Paulo H Ferreira, Hengxing Zhou, Shiqing Feng, Manuela L Ferreira

**Affiliations:** 1 Department of Orthopaedics, Qilu Hospital of Shandong University, Shandong University Centre for Orthopaedics, Advanced Medical Research Institute, Cheeloo College of Medicine, Shandong University, Jinan, Shandong 250012 People’s Republic of China; 2 Department of Biostatistics, School of Public Health, Cheeloo College of Medicine, Shandong University, Jinan, Shandong 250012 People’s Republic of China; 3 Sydney Musculoskeletal Health, The Kolling Institute, Faculty of Medicine and Health, University of Sydney, Sydney, NSW Australia; 4 Sydney Medical School, Faculty of Medicine and Health, University of Sydney, Sydney, NSW Australia; 5 Department of Biostatistics, Mailman School of Public Health, Columbia University, New York, USA; 6 Division of Psychology and Mental Health, Faculty of Biology, Medicine and Health, University of Manchester, Manchester, UK; 7 School of Health Sciences, Faculty of Medicine and Health, University of Sydney, Sydney, Australia; 8 Program in Child Health Evaluative Sciences, The Hospital for Sick Children, Toronto, ON Canada; 9 Institute of Health Policy, Management and Evaluation, University of Toronto, Toronto, ON Canada; 10 School of Population and Global Health, The University of Western Australia, Perth, Australia; 11 Centre for Epidemiology and Biostatistics, Melbourne School of Population and Global Health, University of Melbourne, Melbourne, Australia; 12 Nuffield Department of Orthopaedics, Rheumatology and Musculoskeletal Sciences, University of Oxford, Oxford, UK; 13 The Second Hospital of Shandong University, Cheeloo College of Medicine, Shandong University, Jinan, Shandong 250033 People’s Republic of China

**Keywords:** Depression, Chronic pain, Prevalence, Variability, Clinical prediction model, Big data

## Abstract

**Background:**

The prevalence of depression among people with chronic pain remains unclear due to the heterogeneity of study samples and definitions of depression. We aimed to identify sources of variation in the prevalence of depression among people with chronic pain and generate clinical prediction models to estimate the probability of depression among individuals with chronic pain.

**Methods:**

Participants were from the UK Biobank. The primary outcome was a “lifetime” history of depression. The model’s performance was evaluated using discrimination (optimism-corrected C statistic) and calibration (calibration plot).

**Results:**

Analyses included 24,405 patients with chronic pain (mean age 64.1 years). Among participants with chronic widespread pain, the prevalence of having a “lifetime” history of depression was 45.7% and varied (25.0–66.7%) depending on patient characteristics. The final clinical prediction model (optimism-corrected C statistic: 0.66; good calibration on the calibration plot) included age, BMI, smoking status, physical activity, socioeconomic status, gender, history of asthma, history of heart failure, and history of peripheral artery disease. Among participants with chronic regional pain, the prevalence of having a “lifetime” history of depression was 30.2% and varied (21.4–70.6%) depending on patient characteristics. The final clinical prediction model (optimism-corrected C statistic: 0.65; good calibration on the calibration plot) included age, gender, nature of pain, smoking status, regular opioid use, history of asthma, pain location that bothers you most, and BMI.

**Conclusions:**

There was substantial variability in the prevalence of depression among patients with chronic pain. Clinically relevant factors were selected to develop prediction models. Clinicians can use these models to assess patients’ treatment needs. These predictors are convenient to collect during daily practice, making it easy for busy clinicians to use them.

**Supplementary Information:**

The online version contains supplementary material available at 10.1186/s12916-024-03388-x.

## Background

Chronic pain is one of the leading causes of disability, affecting more than 30% of people worldwide [1]. Depression is also a leading cause of disability, affecting approximately 5% of adults worldwide [2, 3]. It is generally understood that chronic pain and depression are commonly co-morbid disorders [1, 4]. Indeed, research suggests that chronic pain increases the risk of depression, and depression increases the risk of chronic pain [5, 6]. However, the prevalence of depression among people living with chronic pain remains unclear [1, 7]. Previous studies have reported prevalence estimates ranging from about 15% to 85% [8–10]. There are several possible reasons for such variation in the prevalence of depression among people with chronic pain reported across studies. Firstly, measures of depression and definitions of depression vary considerably across studies. For example, some studies measure current depression, while others measure lifetime depression [10]. Secondly, the extent of pain varied (e.g., regional vs widespread pain). Thirdly, the demographic and health characteristics of the populations sampled varied. For example, people with chronic pain who are female, have additional chronic health conditions, or have a lower socioeconomic status are thought to be at higher risk of depression [11].

A clinical prediction model could calculate the risk of a particular endpoint for individual patients by combining multiple predictors, which could be a useful way to accurately estimate the probability that patients with chronic pain suffer from depression based on their individual characteristics [12]. Recently published methodological papers have provided a framework for the development of valid clinical prediction models [13–15].

The selection of the appropriate dataset is important for the development of a valid clinical prediction model. Among potentially suitable datasets, we selected the UK Biobank dataset for the following reasons. Firstly, at its baseline visit, the UK Biobank recruited about 0.5 million participants across the UK, which provided a large sample size to start a study. Secondly, the “experience of pain” questionnaire (2019–2020) provides a comprehensive assessment of chronic pain, including regional or widespread pain, neuropathic or non-neuropathic pain, and pain location that bothers you most. Thirdly, the validity of the measurement of depression in the “online mental health self-assessment” questionnaire (2016–2017) is supported by a dual approach that includes both secondary care record linkage (i.e., diagnosis by a professional) and self-reporting of symptoms [16]. Using this dataset, we aimed to develop and internally validate clinical prediction models of depression among individuals with chronic pain.

## Methods

### Study sample

This study used data from the UK Biobank. UK Biobank is a large-scale biomedical database, which recruited approximately 500,000 people in the UK at its initial enrollment (from 13 March 2006 to 1 October 2010). Part of these participants received follow-up surveys. For example, about 157,000 participants received the “online mental health self-assessment” questionnaire from 13 July 2016 to 27 July 2017, and about 167,000 participants received the “experience of pain” questionnaire from 9 January 2019 to 18 April 2020 [17]. More details about the UK Biobank can be found in the registry online protocol: http://www.ukbiobank.ac.uk. The North West Multi-centre Ethics Committee granted ethical approval to access data from the UK Biobank, and all participants provided written informed consent.

To define chronic pain, we selected the “experience of pain” questionnaire (2019–2020) rather than the baseline visit (2006–2010) for the following reasons. Firstly, the number of pain types in the “experience of pain” questionnaire was much higher than at the baseline visit (i.e., 15 in the “experience of pain” questionnaire compared with 8 in the baseline visit). Secondly, the “experience of pain” questionnaire collected a number of additional pain-related variables (e.g., neuropathic pain or not, and the pain area that bothers you the most). To match the measurement time of chronic pain and depression, the analysis sample was restricted to participants who reported having pain for more than 5 years in the “experience of pain” questionnaire (2019–2020) and completed the “online mental health self-assessment” questionnaire (2016–2017). Based on the International Classification of Diseases 11th Revision definitions for chronic pain and the data availability of UK Biobank, chronic pain was classified as widespread pain (through the question “have you experienced pain or discomfort all over the body?”) and regional pain (i.e., leg pain, chest pain, feet pain, hand pain, arm pain, knee pain, hip pain, stomach or abdominal pain, back pain, neck or shoulder pain, facial pain, and headache) [18].

Although previous literature suggested that multisite pain is strongly related to mood disorders and played an important role in the development of chronic pain, UK Biobank has created a new question, “the pain area that bothers you the most,” in consideration of the fact that many people have multiple pains [19, 20]. Therefore, we included the pain area that bothers you the most as one of the predictors. We also collected the nature of pain (neuropathic and non-neuropathic pain) as one pain-related characteristic [21]. Details for defining pain can be found in Supplementary A.

### Outcomes

We followed the framework that the UK Biobank team proposed to define the depression [16]. The primary outcome was a “lifetime” history of depression rather than present depression, because many mental disorders (e.g., depression) can fluctuate. By including those with a “lifetime” history, we are more likely to more comprehensively capture those with the condition. The dual approach was used to define a “lifetime” history of depression, which included both secondary care record linkage (i.e., diagnosed by a professional) and self-report of symptoms through the Composite International Diagnostic Interview-Short Form (CIDI-SF), depression module, lifetime version. CIDI-SF is a simplified version of its full version CIDI [22] which is a fully structured diagnostic interview, and one previous validation study showed CIDI-SF had comparable accuracy for diagnosing major depressive episodes when compared to CIDI [23]. Two reasons justified the choice of the dual approach: firstly, traditional full-version diagnostic interview is too expensive to be implemented in a cohort with a large sample size (e.g., UK Biobank). Secondly, secondary care record linkage can fail to identify patients with less severe illnesses as these patients are less likely to seek help from the professional compared with patients with more severe illnesses [24] Through this dual approach, all participants were classified as having no “lifetime” history of depression, having a “lifetime” history of subthreshold depressive symptoms, and having a “lifetime” history of depression.

Following the framework that the UK Biobank team proposed, the secondary outcome was present depression [16]. It is worth noting that the UK Biobank team identified present depression among participants with a history of depression, but did not provide clear justification for this approach. Readers should be aware of this point when interpreting the results. Present depression was defined through the Patient Health Questionnaire 9-question version (PHQ-9). PHQ-9 is a validated tool that included nine short screening questionnaires and is widely used in screening for depression [25].

The detailed algorithms and the corresponding R code to define the above outcome were provided by the official group, as available at https://data.mendeley.com/datasets/kv677c2th4/3.

### Covariates

Previous systematic reviews have identified factors that are known to increase risk of depression [11, 26, 27]. Based on these findings and data availability in the UK Biobank and in daily practice, we consider the following variables as covariates: demographic characteristics (age, gender, ethnicity, and Townsend deprivation score which reflected socioeconomic status), body mass index (BMI), lifestyle behaviors (smoking status, alcohol consumption, and physical activity), comorbidities as identified in the recent international consensus on the definition of multimorbidity for research purposes (i.e., stroke, coronary artery disease, heart failure, peripheral artery disease, diabetes, Addison’s disease, cystic fibrosis, chronic obstructive pulmonary disease, asthma, Parkinson’s disease, epilepsy, multiple sclerosis, paralysis, solid organ cancers, hematological cancers, metastatic cancers, dementia, schizophrenia, connective tissue disease, chronic liver disease, inflammatory bowel disease, chronic kidney disease, end-stage kidney disease, and HIV/AIDS) [28], and regular opioid use. For participants with chronic regional pain, nature of pain, and pain location that bothers you most were also added. Definition details could be found in Supplementary B. Other pain severity-related variables were not included as predictors due to the concerns with the potential measurement bias. For example, pain intensity was measured through the question “Thinking about the last 24 hours, how would you rate your pain on a 0-10 scale, where 0 is ‘no pain’ and 10 is ‘pain’ as bad as it could be,” which may not align with the timeline of when patients completed the mental health questionnaire.

### Statistical analysis

Baseline characteristics for participants with chronic pain were shown by depression status. Overall and subgroup prevalence of having: (1) a “lifetime” history of depression among participants with chronic widespread pain; (2) a “lifetime” history of depression among participants with chronic regional pain; (3) present depression among participants with chronic widespread pain; (4) present depression among participants with chronic regional pain were provided. Subgroup analyses were performed based on the “one covariate at a time” principle by each of the variables mentioned in the covariates section. Wald statistic was used to assess whether the prevalence differed by each covariate [29].

Prediction models (through logistic regression) to estimate the probability of depression for individuals with chronic pain were developed. The choice of logistic regression was based on its ease of understanding and communication, as well as its ability to handle binary outcomes [30]. To ensure precise predictions and prevent overfitting, the maximum number of candidate predictor parameters was estimated based on the criteria proposed (details in Supplementary C) by Riley et al. [31]. To minimize the influence of sparse data from binary predictors, we excluded predictors if the number of events in one level of the predictor was less than 10. If the remaining predictors were still more than the estimated maximum number, we excluded predictors with an insignificant Wald statistic. Considering most covariates have a small quantity of missing data (details in Table 1), a single imputation through the transcan function (i.e., a nonlinear additive transformation and imputation function) was used [29].

**Table 1:** Baseline characteristics for participants with chronic pain stratified by depression status

	Having no “lifetime” history of depression	Having a “lifetime” history of subthreshold depressive symptoms	Having a “lifetime” history of depression	Having present depression	Total
Participants	11,137 (45.6)	5317 (21.8)	7951 (32.6)	912 (3.7)	24,405 (100.0)
**Demographic characteristics**					
Age, mean (SD)	65.3 (7.3)	64.3 (7.5)	62.5 (7.3)	59.6 (7.0)	64.1 (7.5)
Gender: male	5036 (45.2)	1963 (36.9)	2229 (28.0)	295 (32.3)	9228 (37.8)
Ethnicity					
White	10,825 (97.2)	5151 (96.9)	7730 (97.2)	877 (96.2)	23,706 (97.1)
Black	69 (0.6)	37 (0.7)	44 (0.6)	9 (1.0)	150 (0.6)
Asian	72 (0.6)	42 (0.8)	44 (0.6)	5 (0.5)	158 (0.6)
Chinese	23 (0.2)	6 (0.1)	14 (0.2)	1 (0.1)	43 (0.2)
Mixed	43 (0.4)	27 (0.5)	44 (0.6)	4 (0.4)	114 (0.5)
Other	51 (0.5)	29 (0.5)	39 (0.5)	9 (1.0)	119 (0.5)
Missing	54 (0.5)	25 (0.5)	36 (0.5)	7 (0.8)	115 (0.5)
Townsend deprivation score					
Most	1192 (10.7)	796 (15.0)	1285 (16.2)	244 (26.8)	3273 (13.4)
Average	3310 (29.7)	1765 (33.2)	2692 (33.9)	331 (36.3)	7767 (31.8)
Least	6622 (59.5)	2750 (51.7)	3959 (49.8)	333 (36.5)	13,331 (54.6)
Missing	13 (0.1)	6 (0.1)	15 (0.2)	4 (0.4)	34 (0.1)
**BMI**					
Obesity	2257 (20.3)	1422 (26.7)	2295 (28.9)	411 (45.1)	5974 (24.5)
Overweight	4617 (41.5)	2076 (39.0)	2954 (37.2)	280 (30.7)	9647 (39.5)
Underweight or normal	4219 (37.9)	1800 (33.9)	2674 (33.6)	214 (23.5)	8693 (35.6)
Missing	44 (0.4)	19 (0.4)	28 (0.4)	7 (0.8)	91 (0.4)
**Lifestyle behaviors**					
Smoking status					
Current	616 (5.5)	450 (8.5)	786 (9.9)	154 (16.9)	1852 (7.6)
Former	4016 (36.1)	2033 (38.2)	3088 (38.8)	326 (35.7)	9137 (37.4)
Never	6475 (58.1)	2820 (53.0)	4053 (51.0)	426 (46.7)	13,348 (54.7)
Missing	30 (0.3)	14 (0.3)	24 (0.3)	6 (0.7)	68 (0.3)
Heavy drinkers	795 (7.1)	395 (7.4)	533 (6.7)	80 (8.8)	1723 (7.1)
Physical activity					
High	3655 (32.8)	1582 (29.8)	2426 (30.5)	251 (27.5)	7663 (31.4)
Moderate	3996 (35.9)	1810 (34.0)	2813 (35.4)	263 (28.8)	8619 (35.3)
Low	1705 (15.3)	941 (17.7)	1457 (18.3)	234 (25.7)	4103 (16.8)
Missing	1781 (16.0)	984 (18.5)	1255 (15.8)	164 (18.0)	4020 (16.5)
**Comorbidities**					
Stroke	161 (1.4)	87 (1.6)	108 (1.4)	13 (1.4)	356 (1.5)
Coronary artery disease	983 (8.8)	557 (10.5)	750 (9.4)	119 (13.0)	2290 (9.4)
Heart failure	75 (0.7)	58 (1.1)	42 (0.5)	9 (1.0)	175 (0.7)
Peripheral artery disease	293 (2.6)	184 (3.5)	258 (3.2)	57 (6.3)	735 (3.0)
Diabetes	524 (4.7)	436 (8.2)	590 (7.4)	145 (15.9)	1550 (6.4)
Addison’s disease	4 (0.0)	9 (0.2)	6 (0.1)	1 (0.1)	19 (0.1)
Cystic fibrosis	3 (0.0)	0 (0.0)	2 (0.0)	1 (0.1)	5 (0.0)
COPD	342 (3.1)	248 (4.7)	383 (4.8)	75 (8.2)	973 (4.0)
Asthma	934 (8.4)	647 (12.2)	1197 (15.1)	189 (20.7)	2778 (11.4)
Parkinson’s disease	23 (0.2)	29 (0.5)	23 (0.3)	2 (0.2)	75 (0.3)
Epilepsy	92 (0.8)	73 (1.4)	107 (1.3)	25 (2.7)	272 (1.1)
Multiple sclerosis	62 (0.6)	50 (0.9)	74 (0.9)	15 (1.6)	186 (0.8)
Paralysis	84 (0.8)	52 (1.0)	77 (1.0)	16 (1.8)	213 (0.9)
Solid organ cancers	1141 (10.2)	558 (10.5)	777 (9.8)	85 (9.3)	2476 (10.1)
Hematological cancers	134 (1.2)	71 (1.3)	86 (1.1)	14 (1.5)	291 (1.2)
Metastatic cancers	245 (2.2)	129 (2.4)	175 (2.2)	19 (2.1)	549 (2.2)
Dementia	10 (0.1)	18 (0.3)	16 (0.2)	2 (0.2)	44 (0.2)
Schizophrenia	2 (0.0)	4 (0.1)	9 (0.1)	4 (0.4)	15 (0.1)
Connective tissue disease	188 (1.7)	135 (2.5)	203 (2.6)	36 (3.9)	526 (2.2)
Chronic liver disease	229 (2.1)	155 (2.9)	259 (3.3)	58 (6.4)	643 (2.6)
Inflammatory bowel disease	211 (1.9)	137 (2.6)	185 (2.3)	20 (2.2)	533 (2.2)
Chronic kidney disease	306 (2.7)	214 (4.0)	259 (3.3)	39 (4.3)	779 (3.2)
End-stage kidney disease	7 (0.1)	5 (0.1)	2 (0.0)	0 (0)	14 (0.1)
HIV/AIDS	1 (0.0)	6 (0.1)	14 (0.2)	5 (0.5)	21 (0.1)
**Regular opioid use**	744 (6.7)	624 (11.7)	1106 (13.9)	221 (24.2)	2575 (10.1)

*Abbreviations*: *COPD* Chronic obstructive pulmonary disease

Data are presented as the number (percentage) of patients unless otherwise indicated

**Fig. 1:**
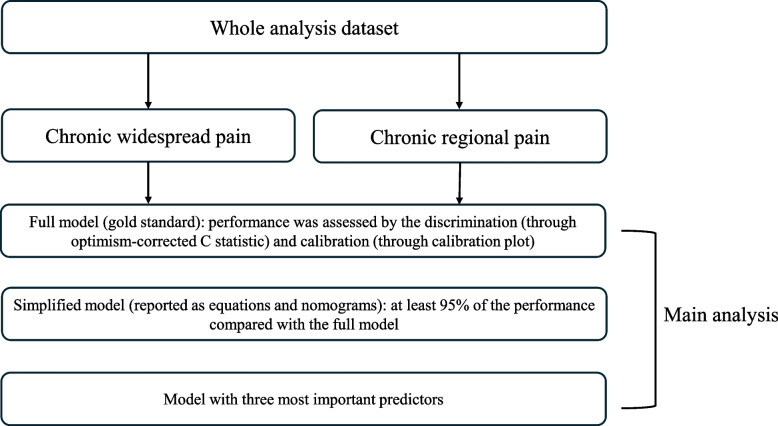
Summary of the modeling strategy

The modeling strategy we used was adapted from Harrell’s Regression Modeling Strategies (detailed in Fig. 1) [29] The full model, including all pre-specified predictors without variable selection, was considered the gold standard. However, clinicians may have insufficient resources (e.g., time) to collect all these predictors. Thus, the simplified model may be needed in daily practice. One significant benefit of Harrell’s simplified model is that it offers varying degrees of parsimony to clinicians based on their specific needs. This is achieved by estimating the contribution of each predictor. In our study, we provide two examples. Firstly, the simplified model (reported as equations and nomograms) has at least 95% of the performance compared with the full model. Secondly, we assume that the clinician only wants to collect the three most important predictors.

Model performance was assessed by the discrimination (through optimism-corrected C statistic) and calibration (through calibration plot) [12]. Optimism is defined as a bias due to overfitting. The bootstrap method is a class of resampling methods that samples a sub-dataset from the original one with replacement. The estimate of the optimism equals the C statistic from the original sample minus the C statistic from the bootstrap sample. In our study, this process was repeated 1000 times to get an average optimism. The final reported optimism-corrected C statistic equals the C statistic from the original sample minus the average optimism [29]. In addition, the C-statistic with the 95% confidence interval using 10-fold cross-validation was provided. We checked whether two continuous variables (age and BMI) should be modeled through splines and the results showed that they can be analyzed through the original form. Based on clinical knowledge and other literature, we assessed the potential interaction between age and ethnicity and the results showed that we did not need to include this interaction term in the model [32]. Details for modeling could be found in Supplementary D.

For chronic regional pain, although one prediction model may not work well for different categories, we did not develop a clinical prediction model for each category as the sample size may be insufficient. To explore the robustness of the prediction model for the overall chronic regional pain, we performed an additional analysis by evaluating its model performance for each category of chronic regional pain.

We reported this study based upon the Strengthening the Reporting of Observational Studies in Epidemiology (STROBE) and Transparent reporting of a multivariable prediction model for individual prognosis or diagnosis (TRIPOD) statement [33, 34]. All statistical analyses were performed in R, version 4.2.2 (R Group for Statistical Computing).

## Results

**Fig. 2:**
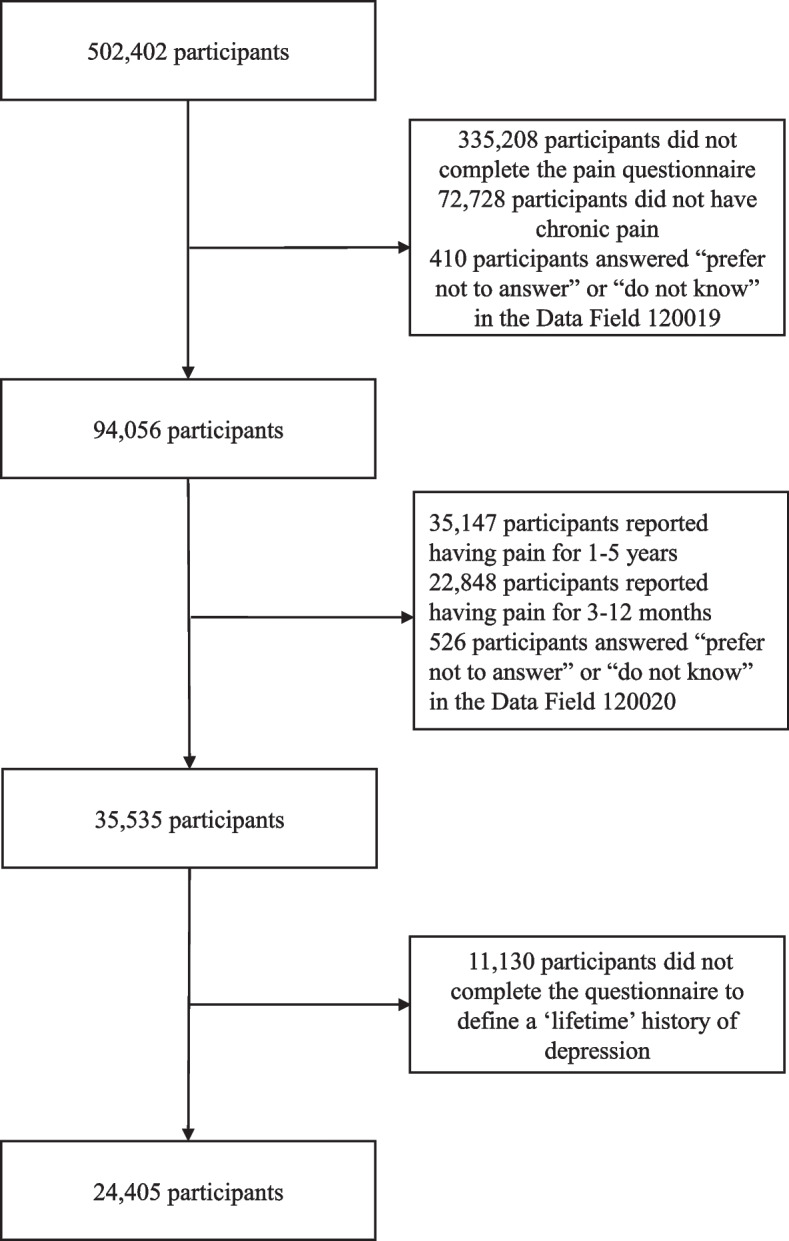
Flowchart of study participants

Of the UK Biobank participants, 24,405 participants with chronic pain were included: 912 (3.7%) had present depression, 7952 (32.6%) had a “lifetime” history of depression, 5317 (21.8%) had a “lifetime” history of subthreshold depressive symptoms, and 11,137 (45.6%) had no “lifetime” history of depression. Figure 2 shows the selection process. Table 1 reports the participants’ baseline characteristics. Among included participants, 9228 (37.8%) were men, the mean (SD) age was 64.1 (7.5) years, and 23,706 (97.1%) were white. Univariate associations of the covariates with depression outcomes could be found in Supplementary E.

### Primary outcome

**Fig. 3:**
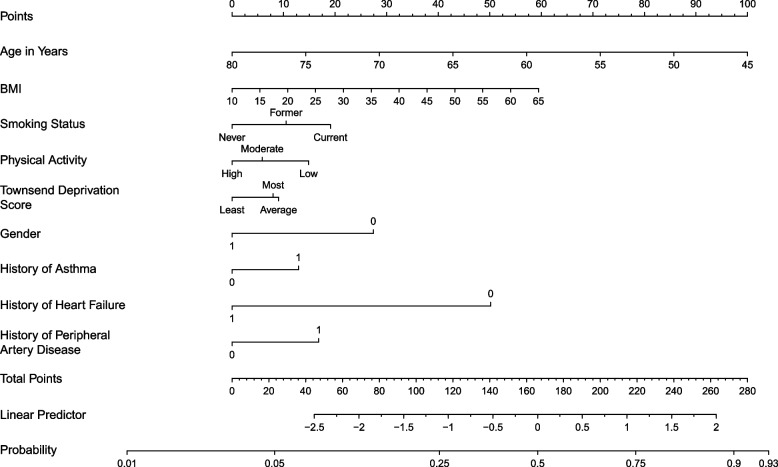
Nomogram for estimating the probability of having a “lifetime” history of depression for individuals with chronic widespread pain. Gender: male—1 and female—0. History of one comorbidity: yes—1 and no—0. Instructions for the use of the nomogram: (1) locate the answer for each predictor, (2) draw a straight line upward to the point axis and record the score, (3) calculate the total score for all predictors and locate the score in the total points axis, (4) draw a straight line downward to the probability axis to estimate the individual’s probability of having a “lifetime” history of depression

Among participants with chronic widespread pain, the prevalence of having a “lifetime” history of depression was 45.7% (1716/3757) (Table 2). Subgroup analyses revealed that the prevalence ranged from 25.0 to 66.7% (Table 2). 26 predictors were included in the initial full prediction model (Supplementary F). The final simplified model (Supplementary G) with nine predictors (age, BMI, smoking status, physical activity, Townsend deprivation score, gender, history of asthma, history of heart failure, and history of peripheral artery disease) was built with its equation in Supplementary H and the nomogram in Fig. 3. The prediction model showed moderate discrimination (optimism-corrected C statistic was 0.66; C statistic from the 10-fold cross-validation: 0.67, 95% confidence interval [CI] 0.65 to 0.69) and good calibration (on the calibration plot) (Supplementary I). Age (as age increases by one year, the odds of having a “lifetime” history of depression decreases: odds ratio [OR] 0.94, 95% CI 0.93 to 0.95), gender (compared to females, males were less likely to have a “lifetime” history of depression: OR 0.56, 95% CI 0.47 to 0.65), and BMI (as the value of BMI increase by one, the odds of having a “lifetime” history of depression also increases: OR 1.02, 95% CI 1.01 to 1.03) were the three most important predictors.

**Table 2:** Overall and subgroup prevalence of depression among participants with chronic pain

	Chronic widespread pain		Chronic regional pain	
	Having a “lifetime” history of depression	Having present depression	Having a “lifetime” history of depression	Having present depression
**Overall**	45.7%, 1716/3757	10.5%, 396/3757	30.2%, 6235/20,648	2.5%, 516/20,648
**Demographic characteristics**				
Age				
45 to 54	61.4%, 333/542	22.3%, 121/542	38.4%, 1047/2729	4.8%, 132/2729
55 to 64	54.4%, 724/1331	14.0%, 186/1331	35.9%, 2531/7050	3.3%, 234/7050
65 or more	35.0%, 659/1884	4.7%, 89/1884	24.4%, 2657/10,869	1.4%, 150/10,869
Gender				
Male	35.9%, 350/975	10.3%, 100/975	22.8%, 1879/8253	2.4%, 195/8253
Female	49.1%, 1366/2782	10.6%, 296/2782	35.1%, 4356/12,395	2.6%, 321/12,395
Ethnicity				
White	45.7%, 1637/3585	10.5%, 377/3585	30.3%, 6093/20,121	2.5%, 500/20,121
Non-white	45.4%, 54/119	10.1%, 12/119	26.6%, 92/346	2.0%, 7/346
Townsend deprivation score				
Most	51.6%, 363/703	16.9%, 119/703	35.9%, 922/2570	4.9%, 125/2570
Average	48.4%, 645/1334	12.1%, 161/1334	31.8%, 2047/6433	2.6%, 170/6433
Least	41.2%, 705/1713	6.8%, 116/1713	28.0%, 3254/11,618	1.9%, 217/11,618
**BMI**				
Obesity	50.6%, 713/1409	14.3%, 201/1409	34.7%, 1582/4565	4.6%, 210/4565
Overweight	43.4%, 603/1388	8.9%, 124/1388	28.5%, 2351/8259	1.9%, 156/8259
Underweight or normal	41.5%, 390/939	7.2%, 68/939	29.5%, 2284/7754	1.9%, 146/7754
**Lifestyle behaviors**				
Smoking status				
Current	55.8%, 198/355	20.8%, 74/355	39.3%, 588/1497	5.3%, 80/1497
Former	45.5%, 663/1458	10.3%, 150/1458	31.6%, 2425/7679	2.3%, 176/7679
Never	43.9%, 847/1930	8.8%, 169/1930	28.1%, 3206/11,418	2.3%, 257/11,418
Alcohol consumption				
Heavy drinker	44.9%, 89/198	12.1%, 24/198	29.1%, 444/1525	3.7%, 56/1525
Not heavy drinker	45.7%, 1627/3559	10.5%, 372/3559	30.3%, 5791/19,123	2.4%, 460/19,123
Physical activity				
High	43.0%, 472/1097	9.0%, 99/1097	29.8%, 1954/6566	2.3%, 152/6566
Moderate	46.6%, 542/1162	8.9%, 103/1162	30.5%, 2271/7457	2.1%, 160/7457
Low	51.9%, 375/723	14.7%, 106/723	32.0%, 1082/3380	3.8%, 128/3380
**Comorbidities**				
Stroke				
Yes	46.0%, 39/63	9.5%, 6/63	27.0%, 79/293	2.4%, 7/293
No	45.7%, 1687/3694	10.6%, 390/3694	30.2%, 6156/20,355	2.5%, 509/20,355
Coronary artery disease				
Yes	42.3%, 218/515	11.3%, 58/515	30.0%, 532/1775	3.4%, 61/1775
No	46.2%, 1498/3242	10.4%, 338/3242	30.2%, 5703/18,873	2.4%, 455/18,873
Heart failure				
Yes	25.0%, 11/44	4.5%, 2/44	23.7%, 31/131	5.3%, 7/131
No	45.9%, 1705/3713	10.6%, 394/3713	30.2%, 6204/20,517	2.5%, 509/20,517
Peripheral artery disease				
Yes	50.6%, 90/178	17.4%, 31/178	30.2%, 168/557	4.7%, 26/557
No	45.4%, 1626/3579	10.2%, 365/3579	30.2%, 6067/20,091	2.4%, 490/20,091
Diabetes				
Yes	50.2%, 227/452	17.7%, 80/452	33.1%, 363/1098	5.9%, 65/1098
No	45.1%, 1489/3305	9.6%, 316/3305	30.0%, 5872/19,550	2.3%, 451/19,550
Addison’s disease				
Yes	60.0%, 3/5	-	21.4%, 3/14	7.1%, 1/14
No	45.7%, 1713/3752	10.6%, 396/3752	30.2%, 6232/20,634	2.5%, 515/20,634
Cystic fibrosis				
Yes	33.3%, 1/3	33.3%, 1/3	50.0%, 1/2	-
No	45.7%, 1715/3754	10.5%, 395/3754	30.2%, 6234/20,646	2.5%, 516/20,646
Chronic obstructive pulmonary disease				
Yes	45.5%, 138/303	12.9%, 39/303	36.6%, 245/670	5.4%, 36/670
No	45.7%, 1578/3454	10.3%, 357/3454	30.0%, 5990/19,978	2.4%, 480/19,978
Asthma				
Yes	53.5%, 386/722	15.1%, 109/722	39.4%, 811/2056	3.9%, 80/2056
No	43.8%, 1330/3035	9.5%, 287/3035	29.2%, 5424/18,592	2.3%, 436/18,592
Parkinson’s disease				
Yes	44.4%, 8/18	-	26.3%, 15/57	3.5%, 2/57
No	45.7%, 1708/3739	10.6%, 396/3343	30.2%, 6220/20,591	2.5%, 514/20,591
Epilepsy				
Yes	54.5%, 36/66	21.2%, 14/66	34.5%, 71/206	5.3%, 11/206
No	45.5%, 1680/3691	10.3%, 382/3691	30.2%, 6164/20,442	2.5%, 505/20,442
Multiple sclerosis				
Yes	49.3%, 35/59	22.0%, 13/59	30.7%, 39/127	1.6%, 2/127
No	45.5%, 1681/3698	10.4%, 383/3315	30.2%, 6196/20,521	2.5%, 514/20,521
Paralysis				
Yes	55.7%, 34/61	16.4%, 10/61	28.3%, 43/152	3.9%, 6/152
No	45.5%, 1682/3696	10.4%, 386/3696	30.2%, 6192/20,496	2.5%, 510/20,496
Solid organ cancers				
Yes	43.7%, 184/421	9.3%, 39/421	28.9%, 593/2055	2.2%, 46/2055
No	45.9%, 1532/3336	10.7%, 357/3336	30.3%, 5642/18,593	2.5%, 470/18,593
Hematological cancers				
Yes	38.5%, 20/52	9.6%, 5/52	27.6%, 66/239	3.8%, 9/239
No	45.8%, 1696/3705	10.6%, 391/3705	30.2%, 6169/20409	2.5%, 507/20409
Metastatic cancers				
Yes	43.6%, 41/94	8.5%, 8/94	29.5%, 134/455	2.4%, 11/455
No	45.7%, 1675/3663	10.6%, 388/3663	30.2%, 6101/20,193	2.5%, 505/20,193
Dementia				
Yes	44.4%, 8/18	5.6%, 1/18	30.8%, 8/26	3.8%, 1/26
No	45.7%, 1708/3739	10.6%, 395/3739	30.2%, 6227/20,622	2.5%, 515/20,622
Schizophrenia				
Yes	-	-	60.0%, 9/15	26.7%, 4/15
No	45.7%, 1716/3757	10.5%, 396/3757	30.2%, 6226/20,633	2.5%, 512/20,633
Connective tissue disease				
Yes	42.9%, 93/217	11.5%, 25/217	35.6%, 110/309	3.6%, 11/309
No	45.8%, 1623/3540	10.5%, 371/3540	30.1%, 6125/20,339	2.5%, 505/20,339
Chronic liver disease				
Yes	53.5%, 108/202	16.8%, 34/202	34.2%, 151/441	5.4%, 24/441
No	45.2%, 1608/3555	10.2%, 362/3555	30.1%, 6084/20,207	2.4%, 492/20,207
Inflammatory bowel disease				
Yes	46.7%, 57/122	7.4%, 9/122	31.1%, 128/411	2.7%, 11/411
No	45.6%, 1659/3635	10.6%, 387/3635	30.2%, 6107/20,237	2.5%, 505/20,237
Chronic kidney disease				
Yes	38.0%, 78/205	6.3%, 13/205	31.5%, 181/574	4.5%, 26/574
No	46.1%, 1638/3552	10.8%, 383/3552	30.2%, 6054/20,074	2.4%, 490/20,074
End-stage kidney disease				
Yes	66.7%, 4/6	-	25.0%, 2/8	-
No	45.7%, 1716/3751	10.6%, 396/3751	30.2%, 6233/20,640	2.5%, 516/20,640
HIV/AIDS				
Yes	50.0%, 2/4	25.0%, 1/4	70.6%, 12/17	23.5%, 4/17
No	45.7%, 1714/3753	10.5%, 395/3753	30.2%, 6223/20,631	2.5%, 512/20,631
**Regular opioid use**				
Yes	50.2%, 461/919	13.8%, 127/919	41.5%, 645/1555	6.0%, 94/1555
No	44.2%, 1255/2838	9.5%, 269/2838	29.3%, 5590/19,093	2.2%, 422/19,093
**Nature of pain**				
Neuropathic pain	-	-	38.3%, 1582/4135	4.3%, 178/4135
Non-neuropathic pain	-	-	28.2%, 4653/16,513	2.0%, 338/16,513
**Pain location that bothers you most**				
Leg pain	-	-	31.4%, 285/907	2.6%, 24/907
Chest pain	-	-	36.0%, 81/225	6.2%, 14/225
Feet pain	-	-	29.7%, 483/1629	2.6%, 42/1629
Hand pain	-	-	31.7%, 451/1421	1.9%, 27/1421
Arm pain	-	-	30.7%, 108/352	2.6%, 9/352
Knee pain	-	-	28.4%, 784/2765	2.1%, 58/2765
Hip pain	-	-	31.8%, 473/1487	2.4%, 36/1487
Stomach or abdominal pain	-	-	37.4%, 293/784	3.8%, 30/784
Back pain	-	-	30.2%, 1376/4552	3.1%, 141/4552
Neck or shoulder pain	-	-	33.7%, 863/2562	2.5%, 63/2562
Facial pain	-	-	36.0%, 71/197	2.5%, 5/197
Headache	-	-	38.7%, 402/1040	3.8%, 39/1040

**Fig. 4:**
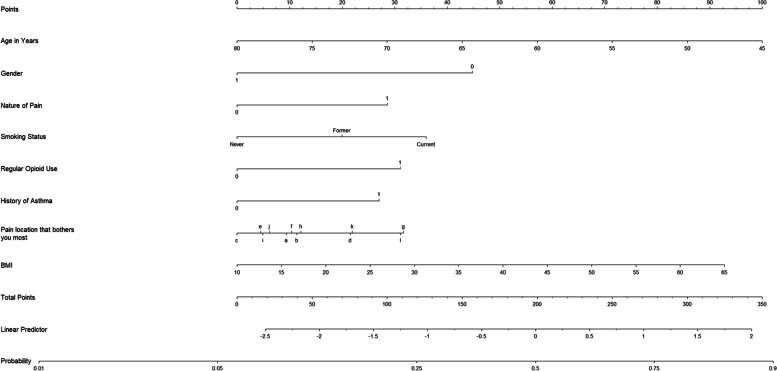
Nomogram for estimating the probability of having a “lifetime” history of depression for individuals with chronic regional pain. Gender: male—1 and female—0. Nature of pain: neuropathic pain—1 and non-neuropathic pain—0. Regular opioid use: yes—1 and no—0. History of asthma: yes—1 and no—0. Pain location that bothers you most: arm pain—a, back pain—b, chest pain—c, facial pain—d, feet pain—e, hand pain—f, headache—g, hip pain—h, knee pain—i, leg pain—j, neck or shoulder pain—k, and stomach or abdominal pain—l. Instructions for the use of the nomogram: (1) locate the answer for each predictor, (2) draw a straight line upward to the point axis and record the score, (3) calculate the total score for all predictors and locate the score in the total points axis, (4) draw a straight line downward to the probability axis to estimate the individual’s probability of having a “lifetime” history of depression

Among participants with chronic regional pain, the prevalence of having a “lifetime” history of depression was 30.2% (6235/20,648) (Table 2). Subgroup analyses revealed that the prevalence ranged from 21.4 to 70.6% (Table 2). Thirty predictors were included in the initial full prediction model (Supplementary F). The final simplified model (Supplementary G) with eight predictors (age, gender, nature of pain, smoking status, regular opioid use, history of asthma, pain location that bothers you most, and BMI) was built with its equation in Supplementary H and the nomogram in Fig. 4. The prediction model showed moderate discrimination (optimism-corrected C statistic was 0.65; C statistic from the 10-fold cross-validation: 0.66, 95% CI 0.65 to 0.66) and good calibration (on the calibration plot) (Supplementary I). Age (as age increases by one year, the odds of having a “lifetime” history of depression decreases: OR 0.96, 95% CI 0.96 to 0.96), gender (compared to females, males were less likely to have a “lifetime” history of depression: OR 0.53, 95% CI 0.50 to 0.57), and nature of pain (compared with patients with non-neuropathic pain, patients with neuropathic pain were more likely to have a “lifetime” history of depression: OR 1.47, 95% CI 1.36 to 1.58) were the three most important predictors.

### Secondary outcome

Among participants with chronic widespread pain, the prevalence of having present depression was 10.5% (396/3757) (Table 2). Subgroup analyses revealed that the prevalence ranged from 4.5 to 33.3% (Table 2). In total, 13 predictors were included in the initial full prediction model (Supplementary F). The final simplified model (Supplementary G) with seven predictors (age, BMI, smoking status, physical activity, Townsend deprivation score, history of peripheral artery disease, and history of chronic kidney disease) was built with its equation in Supplementary H and the nomogram in Supplementary J. The prediction model showed moderate discrimination (optimism-corrected C statistic was 0.75; C statistic from the 10-fold cross-validation: 0.76, 95% CI 0.74 to 0.79) and good calibration (on the calibration plot) (Supplementary I). Age (as age increases by one year, the odds of having present depression decreases: OR 0.91, 95% CI 0.90 to 0.93), BMI (as the value of BMI increases by one, the odds of having present depression also increases: OR 1.04, 95% CI 1.02 to 1.06), and smoking status (compared to current smokers, both former [OR 0.62, 95% CI 0.44 to 0.86] and never [OR 0.47, 95% CI 0.34 to 0.65] smokers were less likely to have present depression) were the three most important predictors.

Among participants with chronic regional pain, the prevalence of having present depression was 2.5% (516/20,648) (Table 2). Subgroup analyses revealed that the prevalence ranged from 1.4 to 26.7% (Table 2). In total, 17 predictors were included in the initial full prediction model (Supplementary F). The final simplified model (Supplementary G) with 10 predictors (age, BMI, nature of pain, pain location that bothers you most, Townsend deprivation score, regular opioid use, physical activity, smoking status, history of diabetes, and history of chronic obstructive pulmonary disease) was built with its equation in Supplementary H and the nomogram in Supplementary J. The prediction model showed moderate discrimination (optimism-corrected C statistic was 0.74; C statistic from the 10-fold cross-validation: 0.75, 95% CI 0.73 to 0.77) and good calibration (on the calibration plot) (Supplementary I). Age (as age increases by one year, the odds of having present depression decrease: OR 0.93, 95% CI 0.92 to 0.94), BMI (as the value of BMI increases by one, the odds of having present depression also increases: OR 1.06, 95% CI 1.04 to 1.07), and nature of pain (compared with patients with non-neuropathic pain, patients with neuropathic pain were more likely to have present depression: OR 1.71, 95% CI 1.40 to 2.10) were the three most important predictors.

### Additional analyses

For the primary outcome (i.e., a “lifetime” history of depression), the results showed that the model developed for the overall chronic regional pain also worked well for all categories (optimism-corrected C statistics: 0.62 to 0.67) of chronic regional pain except for stomach pain (optimism-corrected C statistic: 0.59). For the secondary outcome (i.e., the present depression), the results showed that the model developed for the overall chronic regional pain worked well for all categories (optimism-corrected C statistics: 0.69 to 0.78) of chronic regional pain except for chest pain (optimism-corrected C statistics: 0.64), feet pain (optimism-corrected C statistics: 0.65), hand pain (optimism-corrected C statistics: 0.66), and headache (optimism-corrected C statistics: 0.60).

## Discussion

### Key results

We found that there was substantial variability in the prevalence of having a “lifetime” history of depression among patients with chronic pain. Among participants with chronic widespread pain, the prevalence of having a “lifetime” history of depression was 45.7%; subgroup analyses indicated that the prevalence ranged from 25.0 to 66.7%.

This study developed and evaluated clinical prediction models to estimate the probability of having a “lifetime” history of depression among patients with chronic pain. Among participants with chronic widespread pain, the final clinical prediction model consisted of nine predictors, including age, BMI, smoking status, physical activity, Townsend deprivation score, gender, history of asthma, history of heart failure, and history of peripheral artery disease. Among participants with chronic regional pain, the final clinical prediction model consisted of eight predictors, including age, gender, nature of pain, smoking status, regular opioid use, history of asthma, pain location that bothers you most, and BMI.

### Comparison with previous studies

Using the terms “chronic pain,” “depression,” and “UK Biobank” in PubMed (from the inception to March 1, 2024), we found 13 studies including chronic pain and depression through the analysis of UK Biobank [19, 20, 35–45]. Of the 13 studies, five focused on genetic information [35, 36, 39, 41, 45], five were association analyses [19, 37, 38, 42, 44], one examined the role of coffee in the association between chronic pain and depression [43], one was a clinical prediction model for the development and spread of chronic pain [20], and one assessed risk factors for facial pain [40]. We also extended the search to clinical prediction models based on other datasets and found no other relevant studies. Therefore, this is the first study to develop prediction models that estimate individuals’ probability of experiencing depression among participants with chronic pain. Our models reported through TRIPOD guidelines, showed moderate discrimination and good calibration.

Although our study could not answer the question of bidirectional causality between chronic pain and depression, readers should bear in mind the complex interplay between chronic pain and depression when interpreting the results. Previous studies have reported the role of depression in the chronicity of pain, especially the nociplastic type of pain (fibromyalgia), and the role of chronic pain in the development of depression [20, 46]. Repeated measurements of both pain and depression could facilitate a deeper exploration into whether chronic pain predisposes patients to depression or vice versa [47].

### Limitations

Several limitations should be noted. Firstly, the difference in the measurement time for chronic pain and depression status might bring bias. Although we restricted the analysis sample to those whose pain duration was more than 5 years, we could not totally exclude the influence of recall bias [48]. We also did not find formal analysis to assess the reliability of this retrospective way to define chronic pain, further studies should be performed to assess the accuracy of the estimate. Secondly, genetic information (e.g., polygenic risk scores) may add additional value in predicting depression among individuals with chronic pain, as previous studies have found the genetic relationship between pain and depression [49, 50]. However, the data we applied for this project did not include genetic data. This should be investigated in further studies. Thirdly, although we did not include the number of pain sites as one of the predictors considering the measurement issue in the relevant questionnaire in the UK Biobank, this variable may provide useful information, which should be collected in future studies with more accurate pain questionnaires. Fourthly, although an external validation would be beneficial, a suitable dataset for comparison with the UK Biobank was not found. Further validation studies that prospectively collect data with the comprehensive assessment of chronic pain and depression status are therefore still needed. Fifthly, participants in this study were from the UK, between the ages of 47 to 80, meaning results may not be generalizable to other countries or age groups. Finally, non-white patients were grouped into one category (i.e., the ethnicity in the analysis was treated as a binary variable: white and non-white) in this study to facilitate analysis. However, this way might mask the differences among these non-white patients, which should be explored in future studies.

### Implications of clinical practice and future research

Results from this study can support clinicians in deciding upon treatment priorities for the patient. Importantly, the predictors included are easily collected by clinicians. To further enhance the model, future researchers should focus on improving the quality of the measurement instruments and look to objective assessment when possible. They should also consider other potentially important predictors to improve the predictive accuracy of the model, such as genetic information. Finally, external validation should take place. As Riley et al. mentioned in their new methodological paper, researchers should focus on the target population and setting in which the model is planned to be implemented, especially when the intended population or setting is different from the one in which the model was developed (e.g., UK Biobank) [14].

## Conclusions

There was substantial variability in the prevalence of depression among patients with chronic pain. Clinically relevant factors were selected to develop prediction models. Clinicians can use these models to assess patients’ treatment needs. These predictors are convenient to collect during daily practice, making it easy for busy clinicians to use them.

### Supplementary Information

**Additional file 1:** **Supplementary A.** Details for defining pain. **Supplementary B.** Details for covariates. **Supplementary C.** Sample size calculation. **Supplementary D.** Details for modeling. **Supplementary E.** Univariate associations of the covariates with depression outcomes. **Supplementary F.** Predictors in the initial full model. **Supplementary G.** Details for model approximation. **Supplementary H.** Equations of the final simplified model. **Supplementary I.** Model performance. **Supplementary J.** Nomograms for secondary outcomes.

## Data Availability

Access to data can be requested via application to the UK Biobank (https://www.ukbiobank.ac.uk/). The study was conducted under UK Biobank project number 80584.

## Abbreviations

BMI: Body mass index
CIDI-SF: Composite International Diagnostic Interview-Short Form
PHQ-9: Patient Health Questionnaire 9-question version
SD: Standard deviation
STROBE: Strengthening the Reporting of Observational Studies in Epidemiology
TRIPOD: Transparent reporting of a multivariable prediction model for individual prognosis or diagnosis
