# Supplementary material for: Variability in the prevalence of depression among adults with chronic pain: UK Biobank analysis through clinical prediction models
Source: BMC Med. 2024 Apr 19;22:167. doi: 10.1186/s12916-024-03388-x (PMC11027372; doi:10.1186/s12916-024-03388-x)
Supplement: Supplementary file 1 — Additional file 1: Supplementary A. Details for defining pain. Supplementary B. Details for covariates. Supplementary C. Sample size calculation. Supplementary D. Details for modeling. Supplementary E. Univariate associations of the covariates with depression outcomes. Supplementary F. Predictors in the initial full model. Supplementary G. Details for model approximation. Supplementary H. Equations of the final simplified model. Supplementary I. Model performance. Supplementary J. Nomograms for secondary outcomes. [file 12916_2024_3388_MOESM1_ESM.docx]

**Supplementary Material**

**Variability in the prevalence of depression among adults with chronic pain: UK Biobank analysis through clinical prediction models**

Lingxiao Chen, Claire E Ashton-James, Baoyi Shi, Maja R Radojčić, David B Anderson, Yujie Chen, David B Preen, John L Hopper, Shuai Li, Minh Bui, Paula R Beckenkamp, Nigel K Arden, Paulo H Ferreira, Hengxing Zhou, Shiqing Feng, Manuela L Ferreira

**Items**

**Supplementary A. Details for defining pain**

**Supplementary B. Details for covariates**

**Supplementary C. Sample size calculation**

**Supplementary D. Details for modeling**

**Supplementary E. Univariate associations of the covariates with depression outcomes**

**Supplementary F. Predictors in the initial full model**

**Supplementary G. Details for model approximation**

**Supplementary H. Equations of the final simplified model**

**Supplementary I. Model performance**

**Supplementary J. Nomograms for secondary outcomes**

**Supplementary A. Details for defining pain**

This part is mainly based on the document of the pain web-based questionnaire for UK Biobank (https://biobank.ndph.ox.ac.uk/showcase/refer.cgi?id=2718).

Pain status was defined from the "experience of pain" self-assessment questionnaire issued in 2019.

All participants who completed this questionnaire were asked “Are you troubled by pain or discomfort, either all the time or on and off, that has been present for more than 3 months? (Data-Field 120019)” Participants who answered yes to this question were defined as having chronic pain.

Participants with chronic pain were asked “How long have you been suffering with this pain or discomfort? (Data-Field 120020)” The length of chronic pain was defined from this question.

Participants with chronic pain were asked “In the last 3 months have you experienced pain or discomfort all over the body? (Data-Field 120021)” Participants who answered yes to this question were defined as having chronic widespread pain. Participants who answered no to this question were defined as having chronic regional pain.

Participants with chronic regional pain were asked “Which one of the pains you have experienced in the last 3 months has bothered you most? (Data-Field 120037)” Pain location that bothers you most was defined from this question.

Participants with chronic regional pain were asked seven questions related to the nature of pain: 1) Thinking about the pain that bothers you most, does the pain have the characteristics of burning? (Data-Field 120046); 2) Thinking about the pain that bothers you most, does the pain have the characteristics of painful cold? (Data-Field 120047); 3) Thinking about the pain that bothers you most, does the pain have the characteristics of electric shocks? (Data-Field 120048); 4) Thinking about the pain that bothers you most, is the pain associated with tingling? (Data-Field 120049); 5) Thinking about the pain that bothers you most, is the pain associated with pins and needles? (Data-Field 120050); 6) Thinking about the pain that bothers you most, is the pain associated with numbness? (Data-Field 120051); 7) Thinking about the pain that bothers you most, is the pain associated with itching? (Data-Field 120052). Participants who answered yes to three or more of these seven questions were defined as having neuropathic pain.

**Supplementary B. Details for covariates**

*Age*

Age was defined at the time that participants completed the mental health questionnaire (Data-Field 53 and Data-Field 20400). Age was coded as a continuous variable.

*Gender*

Gender was coded as a binary variable: male and female (Data-Field 31).

*Ethnicity*

Ethnicity was coded as a categorical variable: White, Black, Asian, Chinese, Mixed, and Other (Data-Field 21000). We grouped Black, Asian, Chinese, Mixed, and Other into non-White to facilitate relevant analyses considering the sample size in these groups are small.

*Townsend deprivation score*

Townsend deprivation score is a composite measure of deprivation based on unemployment, non-car ownership, non-home ownership, and household overcrowding; a negative value represents high socioeconomic status. Each participant is assigned a score corresponding to the output area in which their postcode is located. Based on one previous study conducted by the official group (BJPsych Open 2020; 6(2): e18), the Townsend deprivation score was coded as a categorical variable: most, average, and least (Data-Field 189).

*BMI*

BMI was coded as a continuous variable (Data-Field 21001).

*Smoking status*

Smoking status was coded as a categorical variable: current, former, and never (Data-Field 20116).

*Alcohol consumption*

Based on two previous studies (BMC medicine. 2021;19(1):8 and BMC Public Health. 2021 Feb 24;21(1):190), alcohol consumption was measured as average weekly alcohol units. Red wine (Data-Field 1568), champagne plus white wine (Data-Field 1578), beer plus cider (Data-Field 1588), spirits (Data-Field 1598), and fortified wine (Data-Field 1608) were included. Based on one previous study (Nat Commun. 2019 Nov 19;10(1):5039), one glass for wines, one pint for beer/cider, and one measure for spirits corresponding to 2, 2.5, and 1 unit, respectively. Based on one NICE guideline (CG115), heavy drinking was defined as > 35 units/week for women and > 50 units/week for men. Alcohol consumption was coded as a binary variable: heavy drinking vs non-heavy drinking.

*Physical activity*

Based on one previous study (BMJ Open 2016;6:e010038), physical activity participation was assessed using the International Physical Activity Questionnaire (IPAQ) activity group (low, <10.0; moderate, 10.0-49.9 and high, >=50 excess metabolic equivalent (MET)-hours/week). We used the data from the IPAQ activity group (Data-Field 22032). The calculation methods could be found in BMJ Open 2016;6:e010038. The data was generated as part of UKB Application ID 12184. Physical activity was coded as a categorical variable: low, moderate, and high.

*Comorbidities*

Comorbidities were defined based on the recent international consensus in defining multimorbidity in research. 24 always included conditions were selected. Each condition was coded as a binary variable: yes or no (Data-Field 41202 and Data-Field 41204).

| **Conditions** | **ICD-10** | **Reference** |
| --- | --- | --- |
| Stroke | I60, I61, I63, I64 | BMC Med. 2021 Dec 3;19(1):316. |
| Coronary artery disease | I20 - I25 | J Breast Cancer. 2020 Apr 29;23(3):291-302. |
| Congestive heart failure | I50.0 | Circ Heart Fail. 2009 Jan;2(1):18-24. |
| Peripheral artery disease | I70 - I77, I79 |  |
| Diabetes | E10 - E14 | Diabetes Care. 2008 Feb;31(2):335-9. |
| Addison’s disease | E27.1, E27.2 | BMC Med Inform Decis Mak. 2017 Sep 29;17(1):140. |
| Cystic fibrosis | E84 | Int J Environ Res Public Health. 2019 Jan 4;16(1):119. |
| Chronic obstructive pulmonary disease | J40 - J44, J47 | Lancet Respir Med. 2014 Jan;2(1):54-62. |
| Asthma | J45, J46 | Eur J Epidemiol. 2016 Mar;31(3):325-9. |
| Parkinson’s disease | G20 | J Am Med Dir Assoc. 2022 Oct;23(10):1719.e1-1719.e7. |
| Epilepsy | G40, G41 | Int J Epidemiol. 2018 Aug 1;47(4):1151-1158. |
| Multiple sclerosis | G35 | Eur J Epidemiol. 2019 Dec;34(12):1161-1169. |
| Paralysis | G80 - G83 |  |
| Solid organ cancers | C00 - C41, C45 - C76 |  |
| Haematological cancers | C81 - C96 | J Pers Med. 2022 Aug 11;12(8):1306. |
| Metastatic cancers | C77 - C79 | PLOS One. 2016 Sep 20;11(9):e0162864. |
| Dementia | F00 - F03 | J Am Med Dir Assoc. 2016 Jan;17(1):92.e1-7. |
| Schizophrenia | F20 | Br J Psychiatry. 1999 Dec;175:537-43. |
| Connective tissue disease | M30 - M36 | Int J Rheum Dis. 2018 Nov;21(11):1900-1906. |
| Chronic liver disease | K73 - K76 |  |
| Inflammatory bowel disease | K50, K56 | Gut. 2019 Nov;68(11):1953-1960. |
| Chronic kidney disease | N18 | J Hepatol. 2018 May;68(5):904-911. |
| End-stage renal disease | N18.0 |  |
| HIV/AIDS | B20 - B24 | J Infect. 2017 Jan;74(1):22-28. |

*Regular opioid use*

Regular opioid use was defined through Category 100075 which contains data on any regular treatments taken weekly, monthly, etc. Regular opioid use was coded as a binary variable: yes or no (Data-Field 20003).

Detailed codes: 1140864992, 1140865000, 1140928742, 1141153424, 1140922636, 1140928372, 1140871692, 1140882272, 1140871780, 1140871776, 1141152986, 1140882274, 1140871700, 1141152988, 1140871778, 1140871786, 1140871712, 1140871782, 1140871704, 1141152992, 1140871734, 1140871738, 1141180012, 1141180020, 1140871732, 1141171038, 1141171048, 1141171050, 1141171066, 1141170964, 1141180792, 1141171052, 1141170966, 1141171054, 1140880956, 1140911830, 1140911832, 1140911834, 1140911836, 1141150690, 1141150612, 1141150614, 1141150616, 1141150694, 1141150698, 1141150712, 1141150714.

*Nature of pain*

As mentioned above, the nature of pain was coded as a binary variable: neuropathic and non-neuropathic pain.

*Pain location that bothers you most*

As mentioned above, pain location that bothers you most was coded as a categorical variable: leg pain, chest pain, feet pain, hand pain, arm pain, knee pain, hip pain, stomach or abdominal pain, back pain, neck or shoulder pain, facial pain, and headache.

**Supplementary C. Sample size calculation**

R package pmsampsize (version 1.1.2) was used to calculate the minimum sample size required for the development of a new multivariable prediction model.

Considering previous knowledge did not provide a suitable number of candidate predictor parameters for potential inclusion in the new prediction model and we used existing data to develop the model, we estimated the maximum number of candidate predictor parameters. The level of shrinkage was set as 0.9. The C-statistic was set as 0.7. The prevalence was set based on the analysis sample.

**C.1 Probability of having a ‘lifetime’ history of depression for individuals with chronic widespread pain (45.7%, 1716/3757)**


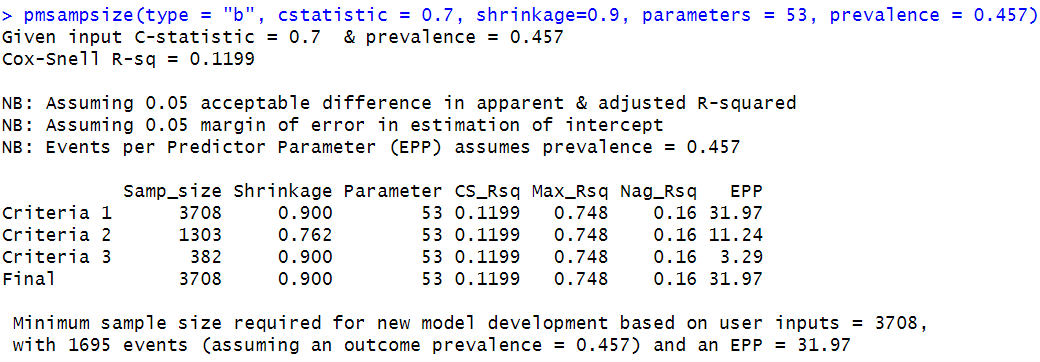


The maximum number of candidate predictor parameters was 53.

**C.2 Probability of having present depression for individuals with chronic widespread pain (10.5%, 396/3757)**


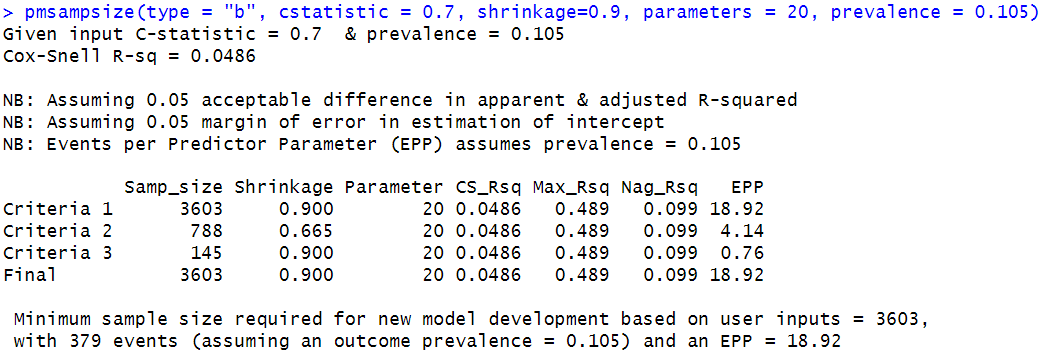


The maximum number of candidate predictor parameters was 20.

**C.3 Probability of having a ‘lifetime’ history of depression for individuals with chronic regional pain (30.2%, 6235/20648)**


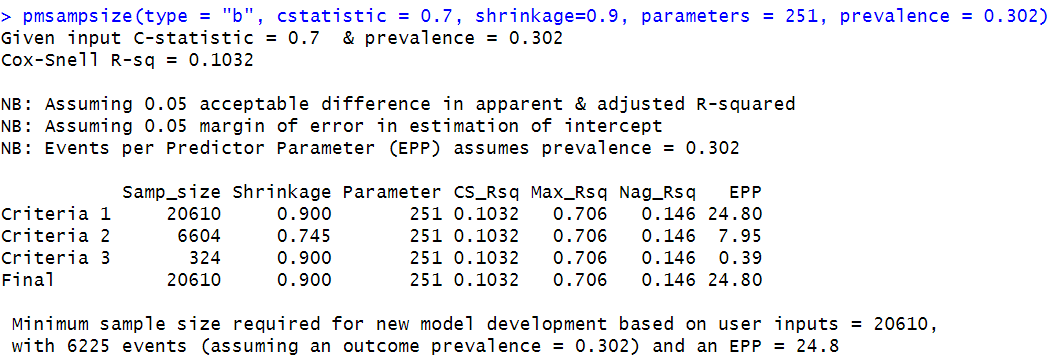


The maximum number of candidate predictor parameters was 251.

**C.4 Probability of having a ‘lifetime’ history of depression for individuals with chronic regional pain (2.5%, 516/20648)**


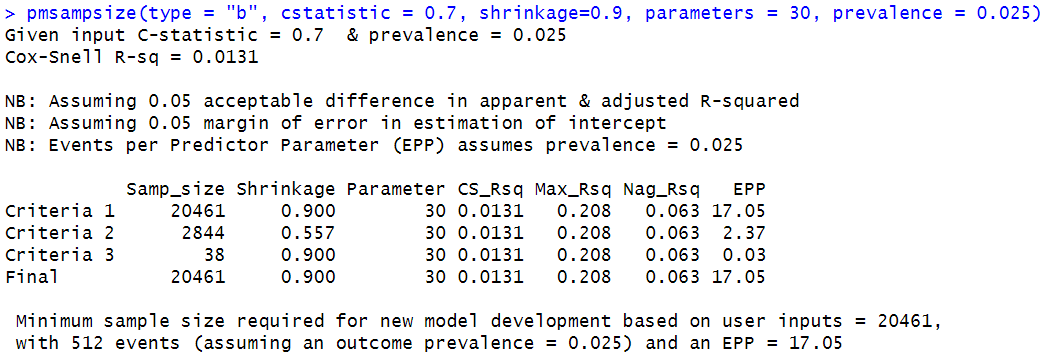


The maximum number of candidate predictor parameters was 30.

**Supplementary D. Details for modeling**

**D.1 Methods to obtain the simplified model**

The first step is to generate the “gold standard” linear predictor from the full model fit (R2=1.0), and the second step is to run ordinary least squares regression through the backward selection method. The final simplified model should have an approximation R2 of at least 0.95.

**D.2 Methods to obtain the model with the three most important predictors**

For selecting the three most important predictors from each model, the importance of each predictor was based on the results from the ordinary least squares regression through the backward selection method (Supplementary F).

**D.3 Methods to model two continuous variables (age and BMI)**

We first checked whether two continuous variables (age and BMI) should be modeled through splines. AIC (smaller is better) was used as a criterion.

**
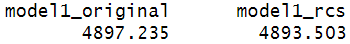
**

**
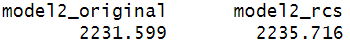
**

**
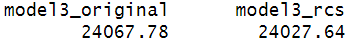
**

**
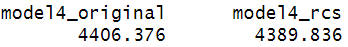
**

We found the AIC was similar between the two modeling ways (continuous vs splines). Thus, we selected the simple way (i.e., model two continuous variables through the original form).

**Supplementary E. Univariate associations of the covariates with depression outcomes**

|  | Chronic widespread pain | | Chronic regional pain | |
| --- | --- | --- | --- | --- |
|  | Having a ‘lifetime’ history of depression | Having present depression | Having a ‘lifetime’ history of depression | Having present depression |
| **Demographic characteristics** |  |  |  |  |
| Age | 0.94 (0.93 to 0.95) | 0.91 (0.90 to 0.92) | 0.96 (0.96 to 0.96) | 0.93 (0.92 to 0.94) |
| Gender |  |  |  |  |
| Female | 1 (reference) | 1 (reference) | 1 (reference) | 1 (reference) |
| Male | 0.58 (0.50 to 0.67) | 0.96 (0.76 to 1.22) | 0.54 (0.51 to 0.58) | 0.91 (0.76 to 1.09) |
| Ethnicity |  |  |  |  |
| Non-white | 1 (reference) | 1 (reference) | 1 (reference) | 1 (reference) |
| White | 1.01 (0.70 to 1.46) | 1.05 (0.57 to 1.92) | 1.20 (0.94 to 1.52) | 1.25 (0.59 to 2.65) |
| Townsend deprivation score |  |  |  |  |
| Most | 1 (reference) | 1 (reference) | 1 (reference) | 1 (reference) |
| Average | 0.88 (0.73 to 1.05) | 0.67 (0.52 to 0.87) | 0.84 (0.76 to 0.92) | 0.52 (0.41 to 0.66) |
| Least | 0.65 (0.55 to 0.78) | 0.36 (0.27 to 0.47) | 0.69 (0.63 to 0.76) | 0.36 (0.29 to 0.45) |
| **BMI** | 1.03 (1.02 to 1.04) | 1.06 (1.05 to 1.08) | 1,02 (1.01 to 1.03) | 1.08 (1.07 to 1.10) |
| **Lifestyle behaviors** |  |  |  |  |
| Smoking status |  |  |  |  |
| Current | 1 (reference) | 1 (reference) | 1 (reference) | 1 (reference) |
| Former | 0.67 (0.53 to 0.84) | 0.43 (0.32 to 0.58) | 0.72 (0.64 to 0.80) | 0.41 (0.32 to 0.54) |
| Never | 0.63 (0.50 to 0.79) | 0.36 (0.27 to 0.49) | 0.60 (0.54 to 0.68) | 0.40 (0.31 to 0.52) |
| Alcohol consumption |  |  |  |  |
| Not heavy drinker | 1 (reference) | 1 (reference) | 1 (reference) | 1 (reference) |
| Heavy drinker | 0.97 (0.73 to 1.29) | 1.18 (0.76 to 1.83) | 0.95 (0.84 to 1.06) | 1.55 (1.17 to 2.05) |
| Physical activity |  |  |  |  |
| High | 1 (reference) | 1 (reference) | 1 (reference) | 1 (reference) |
| Moderate | 1.16 (0.98 to 1.37) | 0.98 (0.73 to 1.31) | 1.03 (0.96 to 1.11) | 0.93 (0.74 to 1.16) |
| Low | 1.43 (1.18 to 1.72) | 1.73 (1.29 to 2.32) | 1.11 (1.02 to 1.22) | 1.66 (1.31 to 2.11) |
| **Comorbidities** |  |  |  |  |
| Stroke |  |  |  |  |
| No | 1 (reference) | 1 (reference) | 1 (reference) | 1 (reference) |
| Yes | 1.01 (0.62 to 1.67) | 0.89 (0.38 to 2.08) | 0.85 (0.66 to 1.10) | 0.95 (0.45 to 2.03) |
| Coronary artery disease |  |  |  |  |
| No | 1 (reference) | 1 (reference) | 1 (reference) | 1 (reference) |
| Yes | 0.85 (0.71 to 1.03) | 1.09 (0.81 to 1.47) | 0.99 (0.89 to 1.10) | 1.44 (1.10 to 1.89) |
| Heart failure |  |  |  |  |
| No | 1 (reference) | 1 (reference) | 1 (reference) | 1 (reference) |
| Yes | 0.39 (0.20 to 0.78) | 0.40 (0.10 to 1.66) | 0.72 (0.48 to 1.07) | 2.22 (1.03 to 4.78) |
| Peripheral artery disease |  |  |  |  |
| No | 1 (reference) | 1 (reference) | 1 (reference) | 1 (reference) |
| Yes | 1.23 (0.91 to 1.66) | 1.86 (1.24 to 2.78) | 1.00 (0.83 to 1.20) | 1.96 (1.31 to 2.93) |
| Diabetes |  |  |  |  |
| No | 1 (reference) | 1 (reference) | 1 (reference) | 1 (reference) |
| Yes | 1.23 (1.01 to 1.50) | 2.03 (1.56 to 2.66) | 1.15 (1.01 to 1.31) | 2.66 (2.04 to 3.48) |
| Addison’s disease |  |  |  |  |
| No | 1 (reference) | - | 1 (reference) | 1 (reference) |
| Yes | 1.79 (0.30 to 10.70) | - | 0.63 (0.18 to 2.26) | 3.01 (0.39 to 23.01) |
| Cystic fibrosis |  |  |  |  |
| No | 1 (reference) | 1 (reference) | 1 (reference) | - |
| Yes | 0.59 (0.05 to 6.56) | 4.25 (0.38 to 47.00) | 2.31 (0.14 to 36.97) | - |
| Chronic obstructive pulmonary disease |  |  |  |  |
| No | 1 (reference) | 1 (reference) | 1 (reference) | 1 (reference) |
| Yes | 0.99 (0.79 to 1.26) | 1.28 (0.90 to 1.83) | 1.35 (1.15 to 1.58) | 2.31 (1.63 to 3.27) |
| Asthma |  |  |  |  |
| No | 1 (reference) | 1 (reference) | 1 (reference) | 1 (reference) |
| Yes | 1.47 (1.25 to 1.73) | 1.70 (1.34 to 2.16) | 1.58 (1.44 to 1.74) | 1.69 (1.32 to 2.15) |
| Parkinson’s disease |  |  |  |  |
| No | 1 (reference) | - | 1 (reference) | 1 (reference) |
| Yes | 0.95 (0.37 to 2.42) | - | 0.83 (0.46 to 1.49) | 1.42 (0.35 to 5.84) |
| Epilepsy |  |  |  |  |
| No | 1 (reference) | 1 (reference) | 1 (reference) | 1 (reference) |
| Yes | 1.44 (0.88 to 2.34) | 2.33 (1.28 to 4.25) | 1.22 (0.91 to 1.63) | 2.23 (1.21 to 4.11) |
| Multiple sclerosis |  |  |  |  |
| No | 1 (reference) | 1 (reference) | 1 (reference) | 1 (reference) |
| Yes | 1.75 (1.04 to 2.95) | 2.45 (1.31 to 4.57) | 1.02 (0.70 to 1.50) | 0.62 (0.15 to 2.52) |
| Paralysis |  |  |  |  |
| No | 1 (reference) | 1 (reference) | 1 (reference) | 1 (reference) |
| Yes | 1.51 (0.91 to 2.51) | 1.68 (0.85 to 3.34) | 0.91 (0.64 to 1.30) | 1.61 (0.71 to 3.66) |
| Solid organ cancers |  |  |  |  |
| No | 1 (reference) | 1 (reference) | 1 (reference) | 1 (reference) |
| Yes | 0.91 (0.75 to 1.12) | 0.85 (0.60 to 1.21) | 0.93 (0.84 to 1.03) | 0.88 (0.65 to 1.20) |
| Haematological cancers |  |  |  |  |
| No | 1 (reference) | 1 (reference) | 1 (reference) | 1 (reference) |
| Yes | 0.74 (0.42 to 1.30) | 0.90 (0.36 to 2.28) | 0.88 (0.66 to 1.17) | 1.54 (0.78 to 3.00) |
| Metastatic cancers |  |  |  |  |
| No | 1 (reference) | 1 (reference) | 1 (reference) | 1 (reference) |
| Yes | 0.92 (0.61 to 1.39) | 0.79 (0.38 to 1.63) | 0.96 (0.79 to 1.18) | 0.97 (0.53 to 1.77) |
| Dementia |  |  |  |  |
| No | 1 (reference) | 1 (reference) | 1 (reference) | 1 (reference) |
| Yes | 0.50 (0.07 to 3.75) | 0.95 (0.37 to 2.42) | 1.03 (0.45 to 2.36) | 1.56 (0.21 to 11.55) |
| Schizophrenia |  |  |  |  |
| No | - | - | 1 (reference) | 1 (reference) |
| Yes | - | - | 3.47 (1.23 to 9.76) | 14.29 (4.54 to 45.03) |
| Connective tissue disease |  |  |  |  |
| No | 1 (reference) | 1 (reference) | 1 (reference) | 1 (reference) |
| Yes | 0.89 (0.67 to 1.17) | 1.11 (0.72 to 1.71) | 1.28 (1.01 to 1.62) | 1.45 (0.79 to 2.66) |
| Chronic liver disease |  |  |  |  |
| No | 1 (reference) | 1 (reference) | 1 (reference) | 1 (reference) |
| Yes | 1.39 (1.05 to 1.85) | 1.79 (1.22 to 2.62) | 1.21 (0.99 to 1.47) | 2.31 (1.51 to 3.51) |
| Inflammatory bowel disease |  |  |  |  |
| No | 1 (reference) | 1 (reference) | 1 (reference) | 1 (reference) |
| Yes | 1.04 (0.73 to 1.50) | 0.67 (0.34 to 1.33) | 1.05 (0.85 to 1.29) | 1.07 (0.59 to 1.97) |
| Chronic kidney disease |  |  |  |  |
| No | 1 (reference) | 1 (reference) | 1 (reference) | 1 (reference) |
| Yes | 0.72 (0.54 to 0.96) | 0.56 (0.32 to 0.99) | 1.07 (0.89 to 1.28) | 1.90 (1.27 to 2.84) |
| End-stage kidney disease |  |  |  |  |
| No | - | - | 1 (reference) | - |
| Yes | - | - | 0.77 (0.16 to 3.82) | - |
| HIV/AIDS |  |  |  |  |
| No | 1 (reference) | 1 (reference) | 1 (reference) | 1 (reference) |
| Yes | 2.83 (0.29 to 27.31) | 1.19 (0.17 to 8.45) | 5.56 (1.96 to 15.78) | 12.09 (3.93 to 37.21) |
| **Regular opioid use** |  |  |  |  |
| No | 1 (reference) | 1 (reference) | 1 (reference) | 1 (reference) |
| Yes | 1.27 (1.09 to 1.47) | 1.53 (1.22 to 1.92) | 1.71 (1.54 to 1.90) | 2.85 (2.26 to 3.58) |
| **Nature of pain** |  |  |  |  |
| Non-neuropathic pain | - | - | 1 (reference) | 1 (reference) |
| Neuropathic pain | - | - | 1.58 (1.47 to 1.70) | 2.15 (1.79 to 2.59) |
| **Pain location** **that bothers you most** |  |  |  |  |
| Arm pain | - | - | 1 (reference) | 1 (reference) |
| Back pain | - | - | 0.98 (0.77 to 1.24) | 1.22 (0.62 to 2.41) |
| Chest pain | - | - | 1.27 (0.89 to 1.81) | 2.53 (1.08 to 5.94) |
| Facial pain | - | - | 1.27 (0.88 to 1.84) | 0.99 (0.33 to 3.00) |
| Feet pain | - | - | 0.95 (0.74 to 1.22) | 1.01 (0.49 to 2.09) |
| Hand pain | - | - | 1.05 (0.82 to 1.35) | 0.74 (0.34 to 1.58) |
| Headache | - | - | 1.42 (1.10 to 1.85) | 1.48 (0.71 to 3.10) |
| Hip pain | - | - | 1.05 (0.82 to 1.36) | 0.95 (0.45 to 1.98) |
| Knee pain | - | - | 0.89 (0.70 to 1.14) | 0.82 (0.40 to 1.66) |
| Leg pain | - | - | 1.04 (0.79 to 1.35) | 1.04 (0.48 to 2.25) |
| Neck or shoulder pain | - | - | 1.15 (0.90 to 1.46) | 0.96 (0.47 to 1.95) |
| Stomach or abdominal pain | - | - | 1.35 (1.03 to 1.76) | 1.52 (0.71 to 3.23) |

All estimates were reported as odds ratio with 95% confidence interval.

**Supplementary F. Predictors in the initial full model**

**F.1 Probability of having a ‘lifetime’ history of depression for individuals with chronic widespread pain: 26 predictors.**

Demographic characteristics (age, gender, ethnicity, and Townsend deprivation score), Body Mass Index (BMI), lifestyle behaviors (smoking status, alcohol consumption, and physical activity), comorbidities (stroke, coronary artery disease, heart failure, peripheral artery disease, diabetes, chronic obstructive pulmonary disease, asthma, epilepsy, multiple sclerosis, paralysis, solid organ cancers, haematological cancers, metastatic cancers, connective tissue disease, chronic liver disease, inflammatory bowel disease, and chronic kidney disease), and regular opioid use.

**F.2 Probability of having present depression for individuals with chronic widespread pain: 13 predictors.**

Demographic characteristics (age, ethnicity, and Townsend deprivation score), Body Mass Index (BMI), lifestyle behaviors (smoking status, and physical activity), comorbidities (peripheral artery disease, diabetes, asthma, epilepsy, multiple sclerosis, chronic liver disease, and chronic kidney disease), and regular opioid use.

**F.3 Probability of having a ‘lifetime’ history of depression for individuals with chronic regional pain: 30 predictors.**

Demographic characteristics (age, gender, ethnicity, and Townsend deprivation score), Body Mass Index (BMI), lifestyle behaviors (smoking status, alcohol consumption, and physical activity), comorbidities (stroke, coronary artery disease, heart failure, peripheral artery disease, diabetes, chronic obstructive pulmonary disease, asthma, Parkinson’s disease, epilepsy, multiple sclerosis, paralysis, solid organ cancers, haematological cancers, metastatic cancers, connective tissue disease, chronic liver disease, inflammatory bowel disease, chronic kidney disease, and HIV/AIDS), regular opioid use, nature of pain, and pain location that bothers you most.

**F.4 Probability of having present depression for individuals with chronic regional pain: 17 predictors.**

Demographic characteristics (age, ethnicity, and Townsend deprivation score), Body Mass Index (BMI), lifestyle behaviors (smoking status, and physical activity), comorbidities (coronary artery disease, peripheral artery disease, diabetes, chronic obstructive pulmonary disease, asthma, epilepsy, chronic liver disease, and chronic kidney disease), regular opioid use, nature of pain, and pain location that bothers you most.

**Supplementary G. Details for model approximation**

**G.1 Probability of having a ‘lifetime’ history of depression for individuals with chronic widespread pain:**

Results from the ordinary least squares regression through the backward selection method


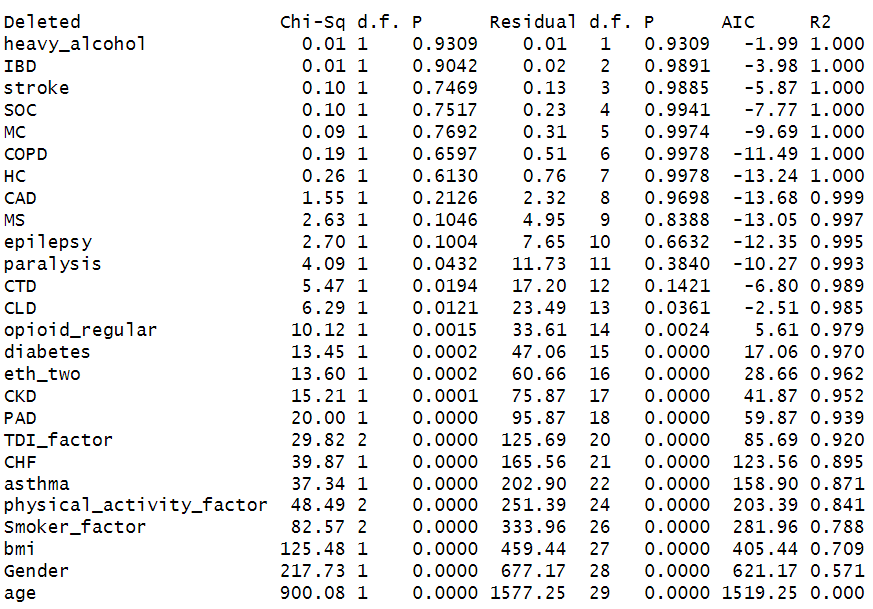


**G.2 Probability of having present depression for individuals with chronic widespread pain:**

Results from the ordinary least squares regression through the backward selection method

**
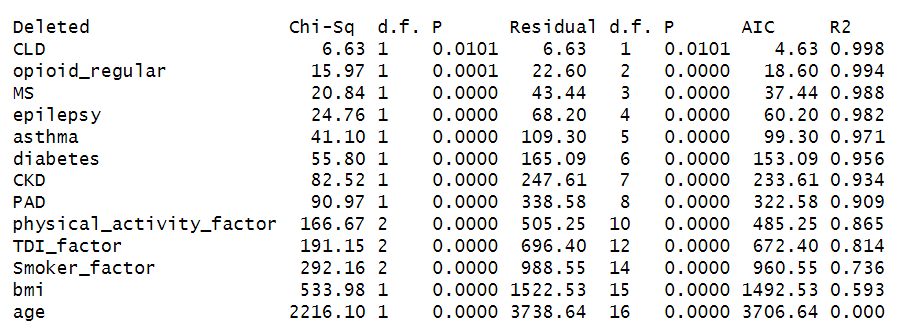
**

**G.3 Probability of having a ‘lifetime’ history of depression for individuals with chronic regional pain:**

Results from the ordinary least squares regression through the backward selection method

**
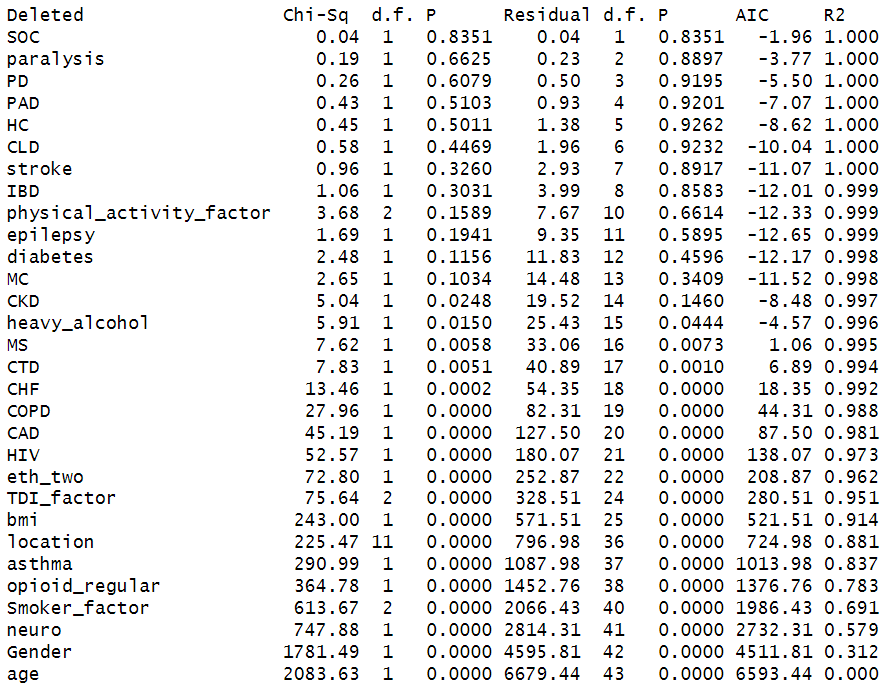
**

**G.4 Probability of having present depression for individuals with chronic regional pain:**

Results from the ordinary least squares regression through the backward selection method

**
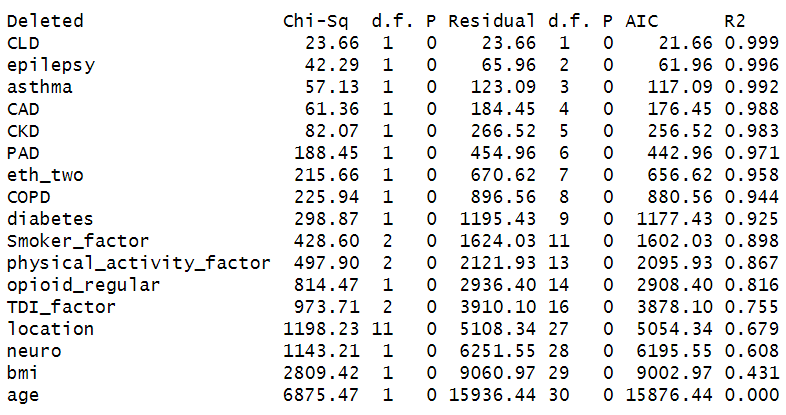
**

**Supplementary H. Equations of the final simplified model**

lp means the linear predictor from the full model.

**H.1 Probability of having a ‘lifetime’ history of depression for individuals with chronic widespread pain:**

Exp (lp)=

3.2241

+ Age × (-0.0589)

+ BMI × (0.0223)

+ Former (smoking status) × (-0.1774)

+ Never (smoking status) × (-0.3935)

+ Moderate (physical activity) × (0.1208)

+ Low (physical activity) × (0.3062)

+ Average (Townsend deprivation score) × (0.0221)

+ Least (Townsend deprivation score) × (-0.1642)

+ Male × (-0.5651)

+ History of asthma × (0.2656)

+ History of heart failure × (-1.0342)

+ History of peripheral artery disease × (0.3469)

**H.2 Probability of having present depression for individuals with chronic widespread pain:**

Exp (lp)=

2.4627

+ Age × (-0.0912)

+ BMI × (0.0507)

+ Former (smoking status) × (-0.5020)

+ Never (smoking status) × (-0.7851)

+ Moderate (physical activity) × (0.1984)

+ Low (physical activity) × (0.5775)

+ Average (Townsend deprivation score) × (-0.0944)

+ Least (Townsend deprivation score) × (-0.5056)

+ History of peripheral artery disease × (0.7828)

+ History of chronic kidney disease × (-0.6627)

**H.3 Probability of having a ‘lifetime’ history of depression for individuals with chronic regional pain:**

Exp (lp)=

1.4293

+ Age × (-0.0397)

+ Male × (-0.6224)

+ Neuropathic pain (nature of pain) × (0.3972)

+ Former (smoking status) × (-0.2225)

+ Never (smoking status) × (-0.4993)

+ Regular opioid use × (0.4315)

+ History of asthma × (0.3756)

+ Back pain (pain location that bothers you most) × (0.0277)

+ Chest pain (pain location that bothers you most) × (-0.1304)

+ Facial pain (pain location that bothers you most) × (0.1687)

+ Feet pain (pain location that bothers you most) × (-0.0690)

+ Hand pain (pain location that bothers you most) × (0.0139)

+ Headache (pain location that bothers you most) × (0.3100)

+ Hip pain (pain location that bothers you most) × (0.0378)

+ Knee pain (pain location that bothers you most) × (-0.0626)

+ Leg pain (pain location that bothers you most) × (-0.0453)

+ Neck or shoulder pain (pain location that bothers you most) × (0.1742)

+ Stomach or abdominal pain (pain location that bothers you most) × (0.3019)

+ BMI × (0.0234)

**H.4 Probability of having present depression for individuals with chronic regional pain:**

Exp (lp)=

-0.4889

+ Age × (-0.0724)

+ BMI × (0.0572)

+ Neuropathic pain (nature of pain) × (0.5544)

+ Back pain (pain location that bothers you most) × (0.3804)

+ Chest pain (pain location that bothers you most) × (0.2570)

+ Facial pain (pain location that bothers you most) × (-0.2258)

+ Feet pain (pain location that bothers you most) × (0.0228)

+ Hand pain (pain location that bothers you most) × (-0.1430)

+ Headache (pain location that bothers you most) × (0.6485)

+ Hip pain (pain location that bothers you most) × (0.1354)

+ Knee pain (pain location that bothers you most) × (-0.0778)

+ Leg pain (pain location that bothers you most) × (-0.0562)

+ Neck or shoulder pain (pain location that bothers you most) × (0.1265)

+ Stomach or abdominal pain (pain location that bothers you most) × (0.4803)

+ Average (Townsend deprivation score) × (-0.3976)

+ Least (Townsend deprivation score) × (-0.5770)

+ Regular opioid use × (0.6514)

+ Moderate (physical activity) × (0.0275)

+ Low (physical activity) × (0.3798)

+ Former (smoking status) × (-0.5063)

+ Never (smoking status) × (-0.5205)

+ History of diabetes × (0.5250)

+ History of chronic obstructive pulmonary disease × (0.6009)

**Supplementary I. Model performance**

**I.1 Probability of having a ‘lifetime’ history of depression for individuals with chronic widespread pain:**

Discrimination


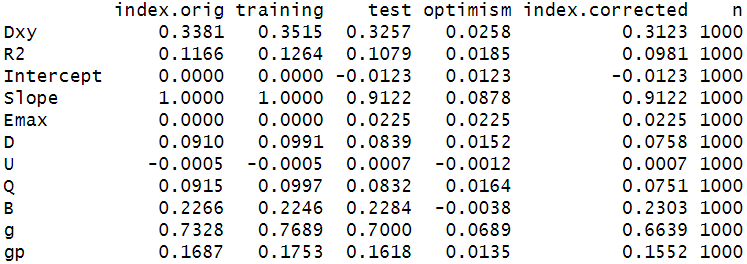


C statistic = (1+Dxy)/2, thus, optimism-corrected C statistic = 0.66

Calibration


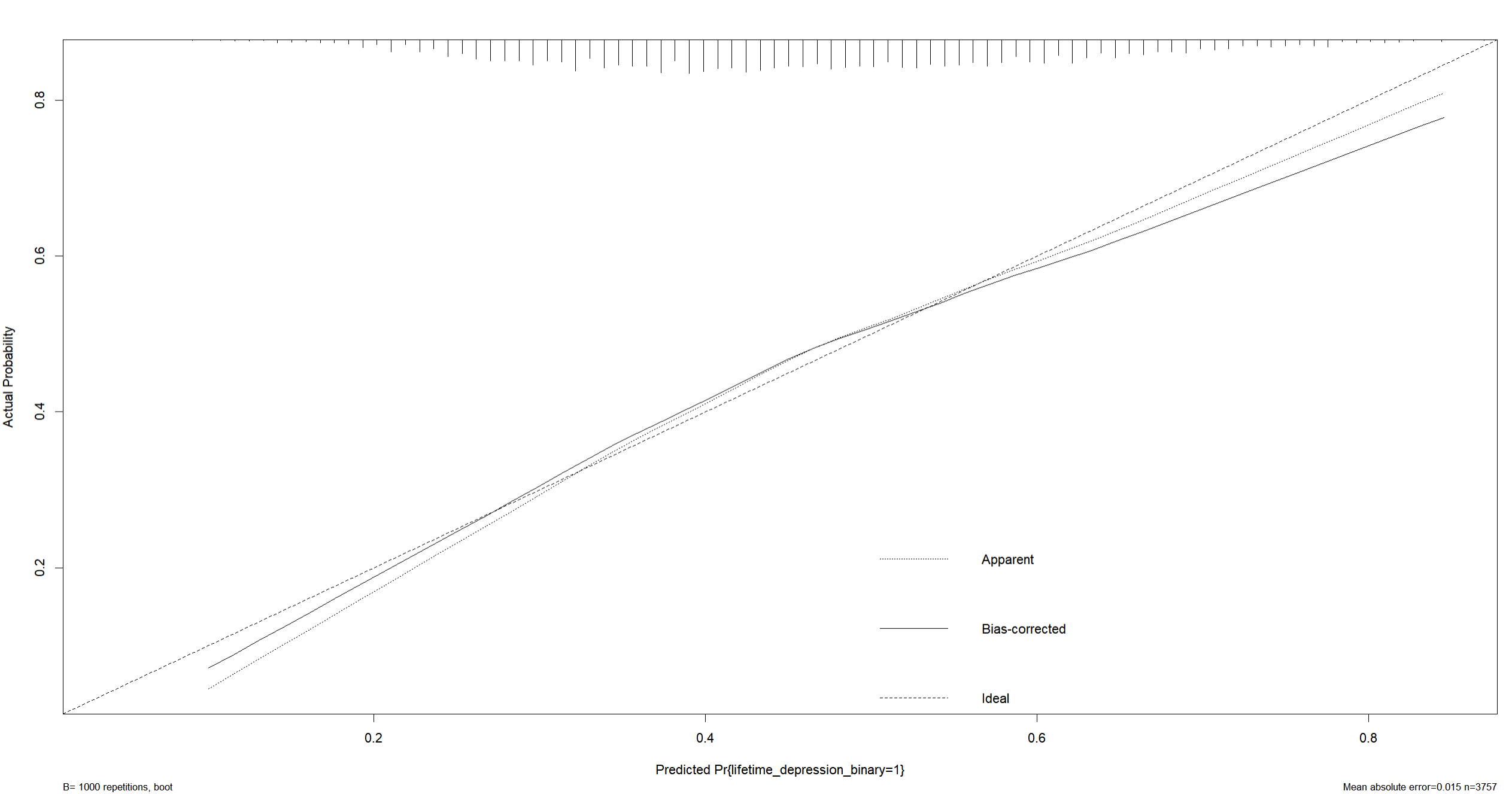


**I.2 Probability of having present depression for individuals with chronic widespread pain:**

Discrimination


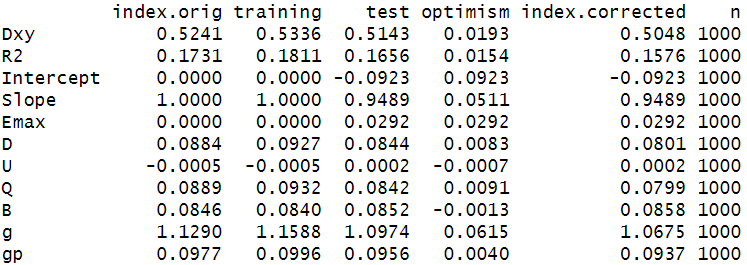


C statistic = (1+Dxy)/2, thus, optimism-corrected C statistic = 0.75

Calibration


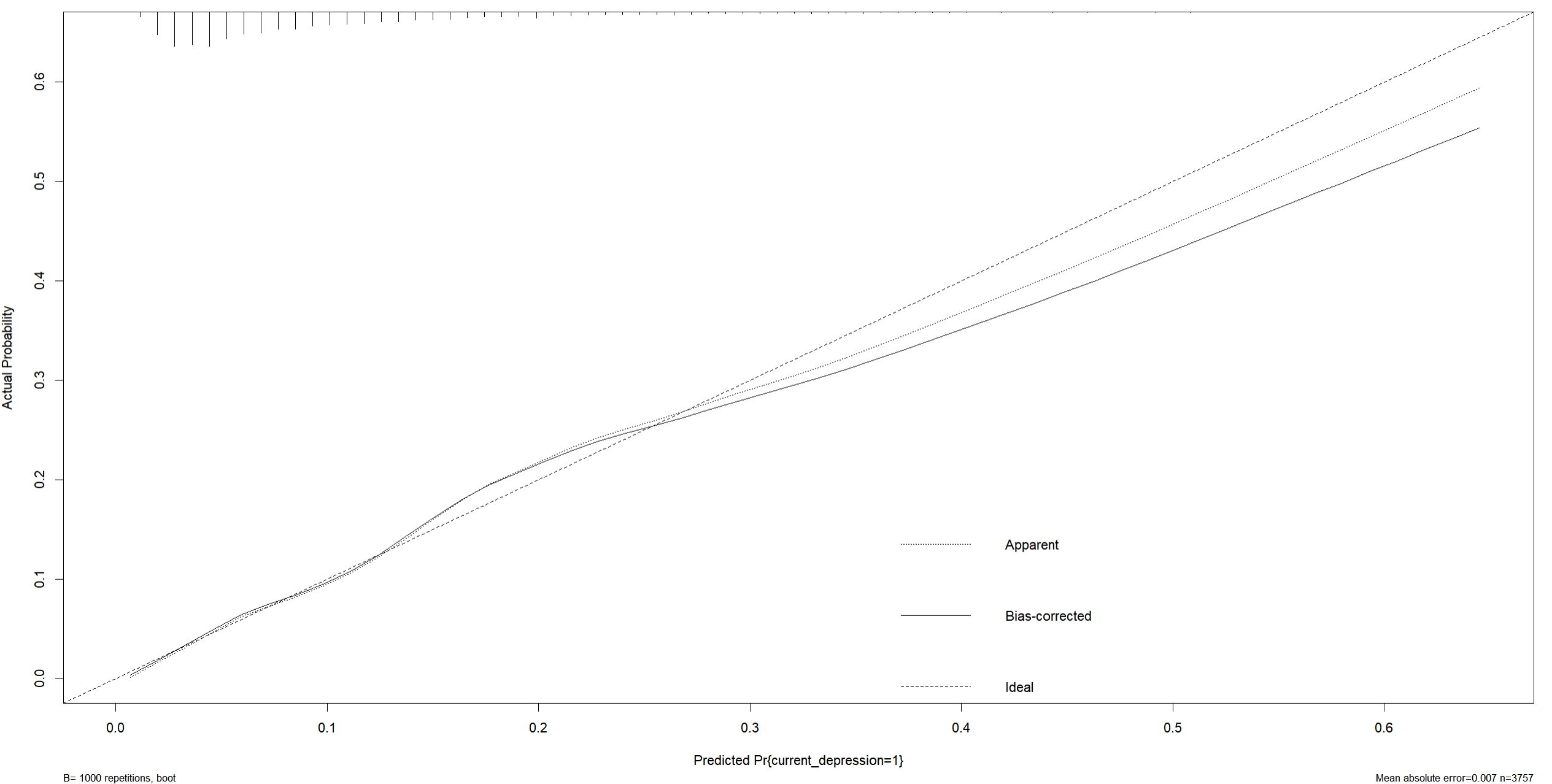


**I.3 Probability of having a ‘lifetime’ history of depression for individuals with chronic regional pain:**

Discrimination


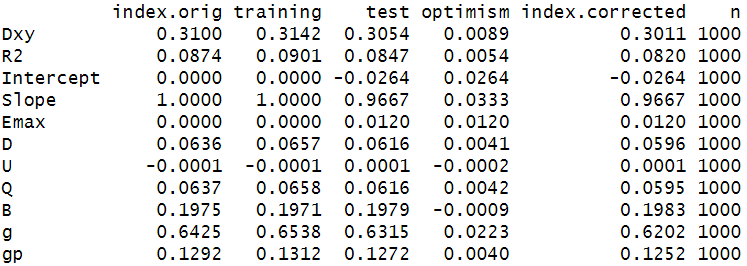


C statistic = (1+Dxy)/2, thus, optimism-corrected C statistic = 0.65

Calibration

**
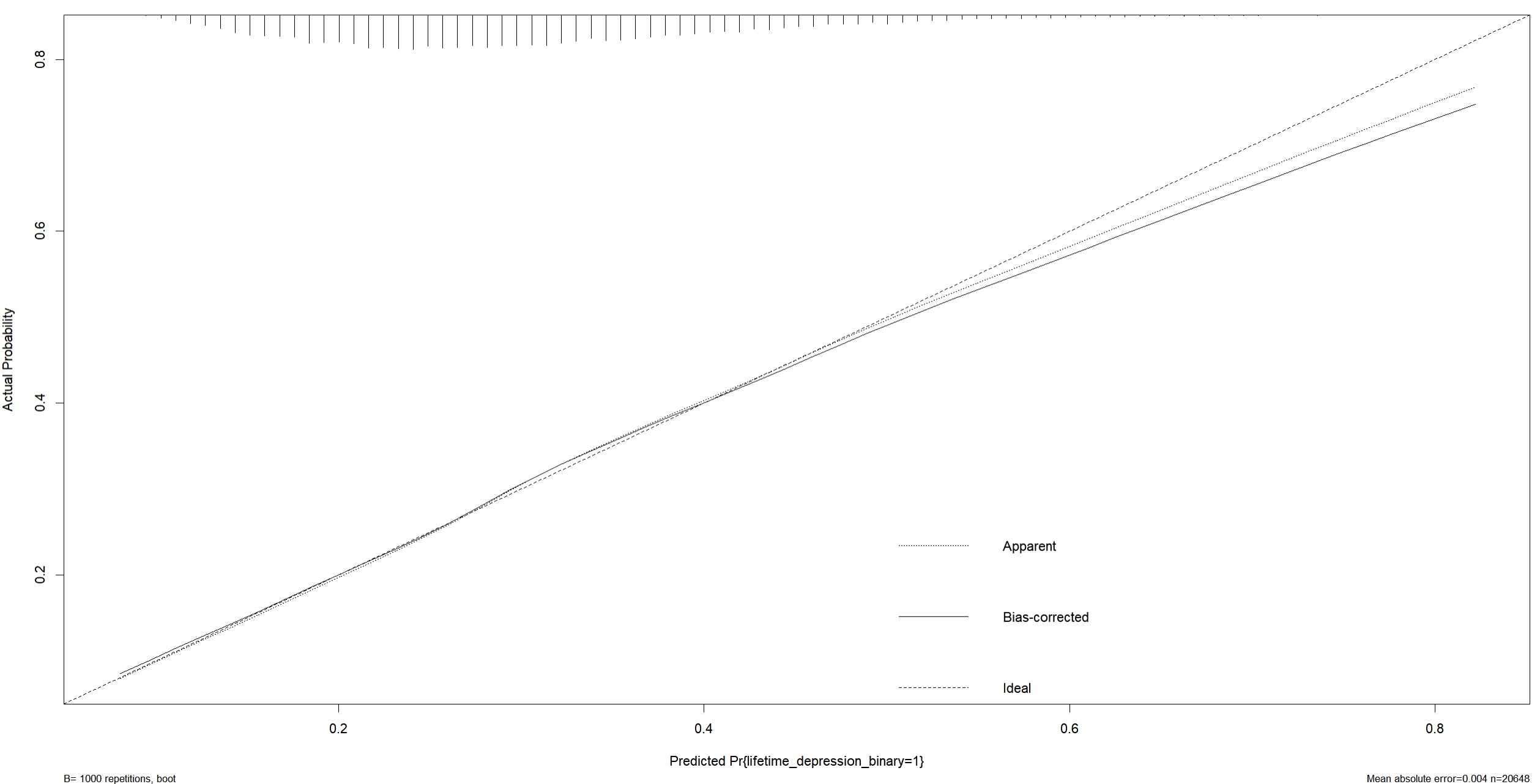
**

**I.4 Probability of having present depression for individuals with chronic regional pain:**

Discrimination


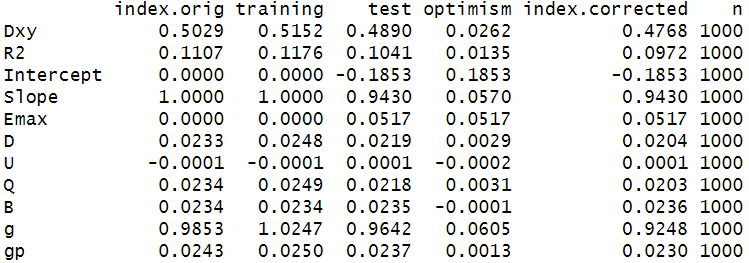


C statistic = (1+Dxy)/2, thus, optimism-corrected C statistic = 0.74

Calibration


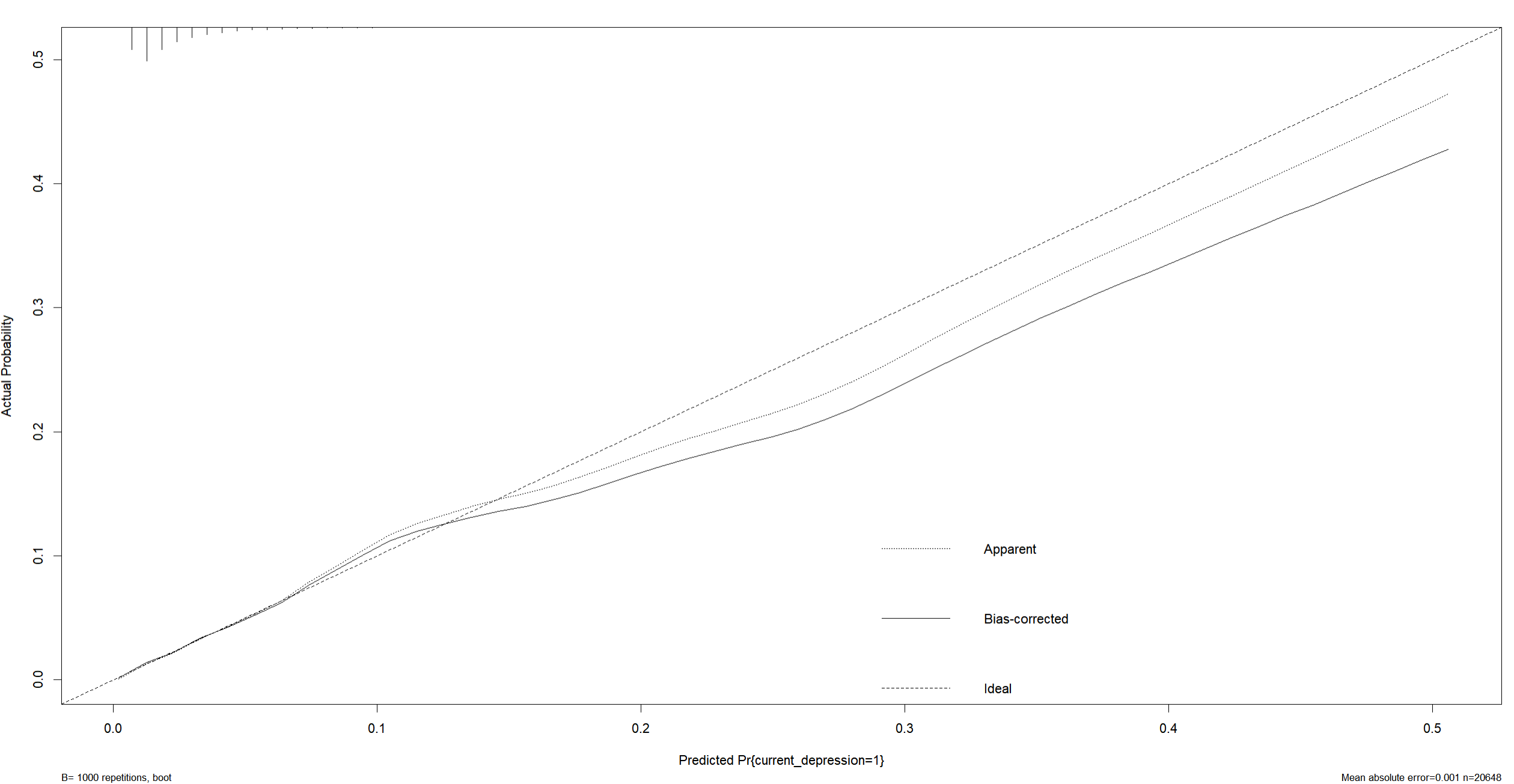


**Supplementary J. Nomograms for secondary outcomes**

**J.1 Nomogram for estimating the probability of having present depression for individuals with chronic widespread pain**


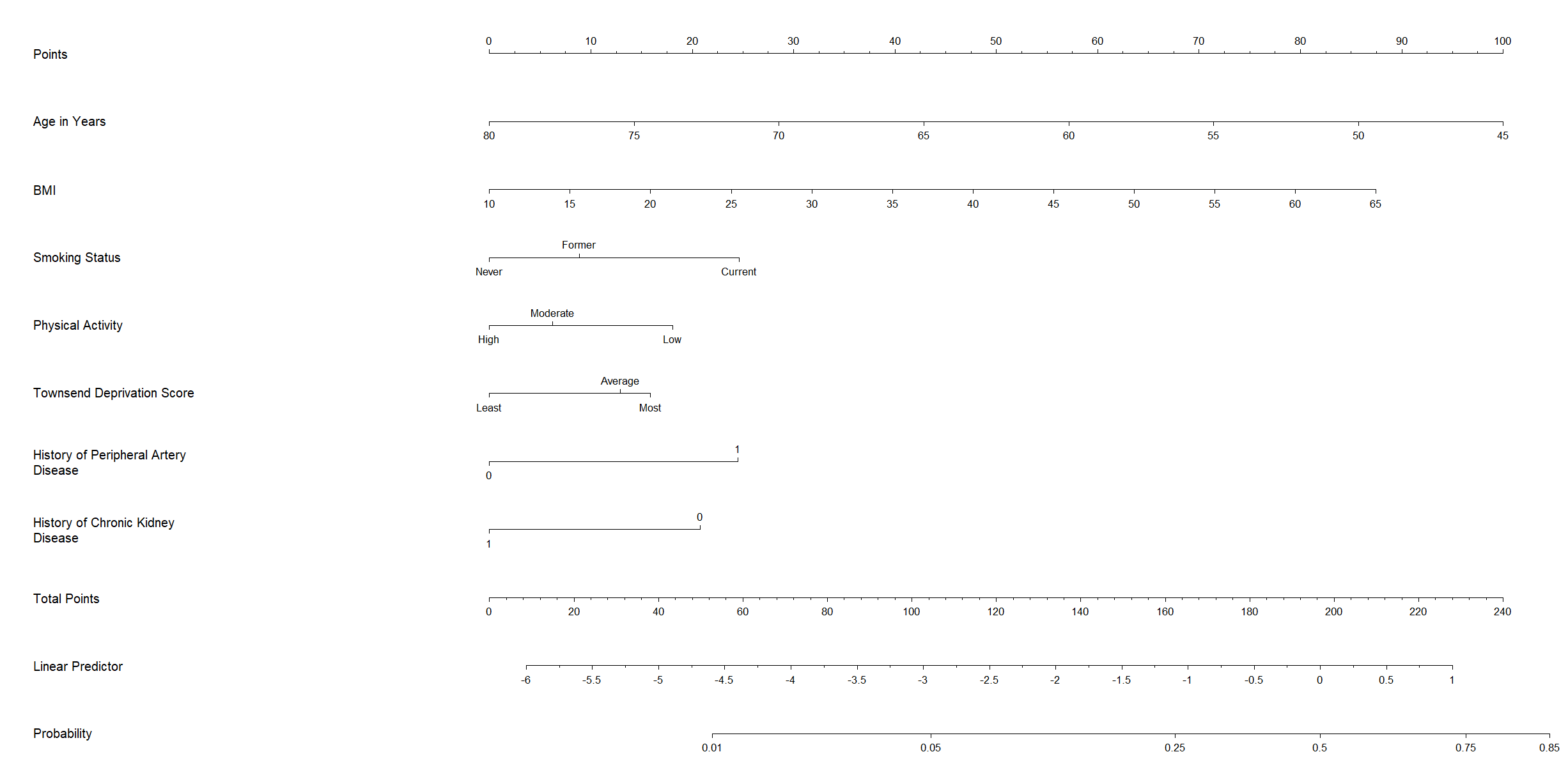


History of one comorbidity: yes-1 and no-0.

Instructions for the use of the nomogram: (1) locate the answer for each predictor, (2) draw a straight line upward to the point axis and record the score, (3) calculate the total score for all predictors and locate the score in the total points axis, (4) draw a straight line downward to the probability axis to estimate the individual’s probability of having present depression.

**J.2 Nomogram for estimating the probability of having present depression for individuals with chronic regional pain**


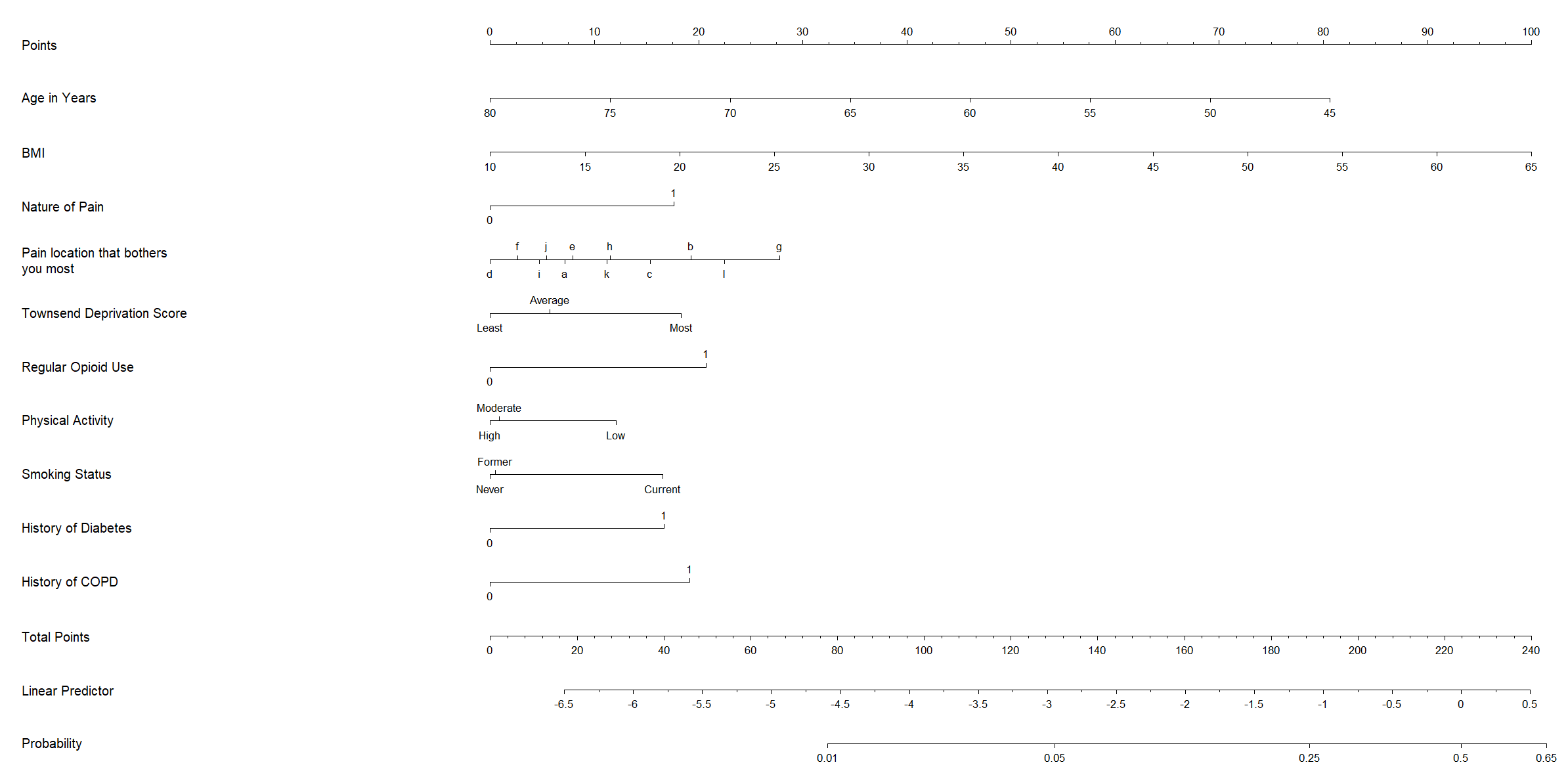


Nature of pain: neuropathic pain-1 and non-neuropathic pain-0. Pain location that bothers you most: arm pain-a, back pain-b, chest pain-c, facial pain-d, feet pain-e, hand pain-f, headache-g, hip pain-h, knee pain-i, leg pain-j, neck or shoulder pain-k, and stomach or abdominal pain-l. Regular opioid use: yes-1 and no-0. History of one comorbidity: yes-1 and no-0.

Instructions for the use of the nomogram: (1) locate the answer for each predictor, (2) draw a straight line upward to the point axis and record the score, (3) calculate the total score for all predictors and locate the score in the total points axis, (4) draw a straight line downward to the probability axis to estimate the individual’s probability of having present depression.
